# Biocatalysts Based on Peptide and Peptide Conjugate Nanostructures

**DOI:** 10.1021/acs.biomac.1c00240

**Published:** 2021-04-12

**Authors:** Ian W. Hamley

**Affiliations:** Department of Chemistry, University of Reading, RG6 6AD Reading, United Kingdom

## Abstract

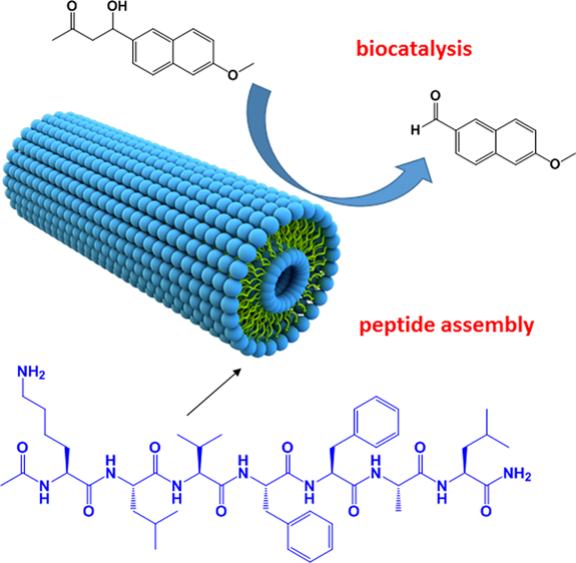

Peptides and their conjugates (to lipids, bulky N-terminals, or other groups) can self-assemble into nanostructures such as fibrils, nanotubes, coiled coil bundles, and micelles, and these can be used as platforms to present functional residues in order to catalyze a diversity of reactions. Peptide structures can be used to template catalytic sites inspired by those present in natural enzymes as well as simpler constructs using individual catalytic amino acids, especially proline and histidine. The literature on the use of peptide (and peptide conjugate) α-helical and β-sheet structures as well as turn or disordered peptides in the biocatalysis of a range of organic reactions including hydrolysis and a variety of coupling reactions (e.g., aldol reactions) is reviewed. The simpler design rules for peptide structures compared to those of folded proteins permit ready *ab initio* design (minimalist approach) of effective catalytic structures that mimic the binding pockets of natural enzymes or which simply present catalytic motifs at high density on nanostructure scaffolds. Research on these topics is summarized, along with a discussion of metal nanoparticle catalysts templated by peptide nanostructures, especially fibrils. Research showing the high activities of different classes of peptides in catalyzing many reactions is highlighted. Advances in peptide design and synthesis methods mean they hold great potential for future developments of effective bioinspired and biocompatible catalysts.

## 1 Introduction

Biocatalysis refers to the enhancement of the rate of reactions stimulated by biological molecules or their components such as proteins and peptides. The development of biocatalysts is of increasing interest due to the possibility to use them in greener and more environmentally friendly processes. The majority of enzymes are biocatalytic proteins, which have evolved to have a diversity of functions in nature.^1−3^ Enzymes have also been harnessed for use in many industries including the production of food and beverages, biofuels, paper, detergents, and others. As well as their functionality *in vivo*, some proteins and peptides have also been shown to have strong activity in organocatalysis, i.e. as catalysts of organic reactions in both aqueous and nonaqueous solvents.

Several classes of peptides including surfactant-like peptides, amyloid peptides, and lipopeptides (a type of peptide amphiphile) can aggregate in aqueous solution into a range of nanostructures depending on intermolecular forces, especially hydrophobic interactions which are balanced by hydrogen-bonding, electrostatic, and π-stacking interactions leading to different self-assembled morphologies. This behavior has been reviewed in detail elsewhere.^4−11^ The present Review is focused on the use of self-assembled peptide structures in biocatalysis. Self-assembled peptide nanostructures can be used to position catalytic residues and/or cofactors in defined positions to enhance catalytic performance under mild aqueous conditions and to permit operation under nonambient conditions. Peptides have advantages as biocatalysts since they are bioderived molecules which can be obtained and purified easily and they enable the design of functional biomolecules using recently established design principles including the control of nanostructure (tertiary structure in the nomenclature of proteins). A variety of self-assembled peptide nanostructures have been used to enhance catalytic activity, including micelles and vesicles in which the peptide often lacks a highly ordered conformation. Peptide coiled-coil aggregate^12−19^ and nanofibril^20−23^ and nanotube^24−29^ structures also have great potential in this respect due to the high degree of internal order, the potential stability of β-sheet and α-helical structures to temperature and pH changes, and the anisotropic presentation of functional (catalytic) residues at high density. Lipidation at the C- or N-terminus is a powerful tool to additionally enhance the stability of peptide structures and to tune peptide self-assembly propensity.^8,10^ Attachment of polymer chains such as PEG (polyethylene glycol) is another means to do this,^30^ although as yet fewer polymer–peptide conjugates have been developed for biocatalysis.

The present Review concerns the use of peptides in biocatalysis, specifically in organocatalysis, and the development of peptides with enzyme-like activities. This topic has been covered in previous reviews.^31−33^ The enzymatic activity of peptide fibrils has been the theme of a focused overview,^34^ as have the catalytic properties of supramolecular gels including those of peptide-based molecules in organocatalysis.^35,36^ The development of small protein (“minimalist”) catalysts has also been reviewed.^37,38^ The present Review covers peptide catalysts and not those created from larger proteins. The cutoff between a long peptide and a short protein (mini-protein) is somewhat arbitrary; the present Review considers peptides with fewer than ca. 100 residues (i.e., a molar mass of ca. 10 kg/mol or less). In addition, this Review does not cover the topics reviewed elsewhere of biocatalytic synthesis of peptides^39−43^ or enzyme-assisted (enzyme-instructed) peptide self-assembly (or disassembly).^44−47^ This Review concerns reactions catalyzed *by* peptide assemblies not catalysis *of* peptide self-assemblies which has been the focus of recent remarkable work by the groups of Ulijn,^45,48−52^ Xu,^53−56^ and others.^57^

**Scheme 1:**
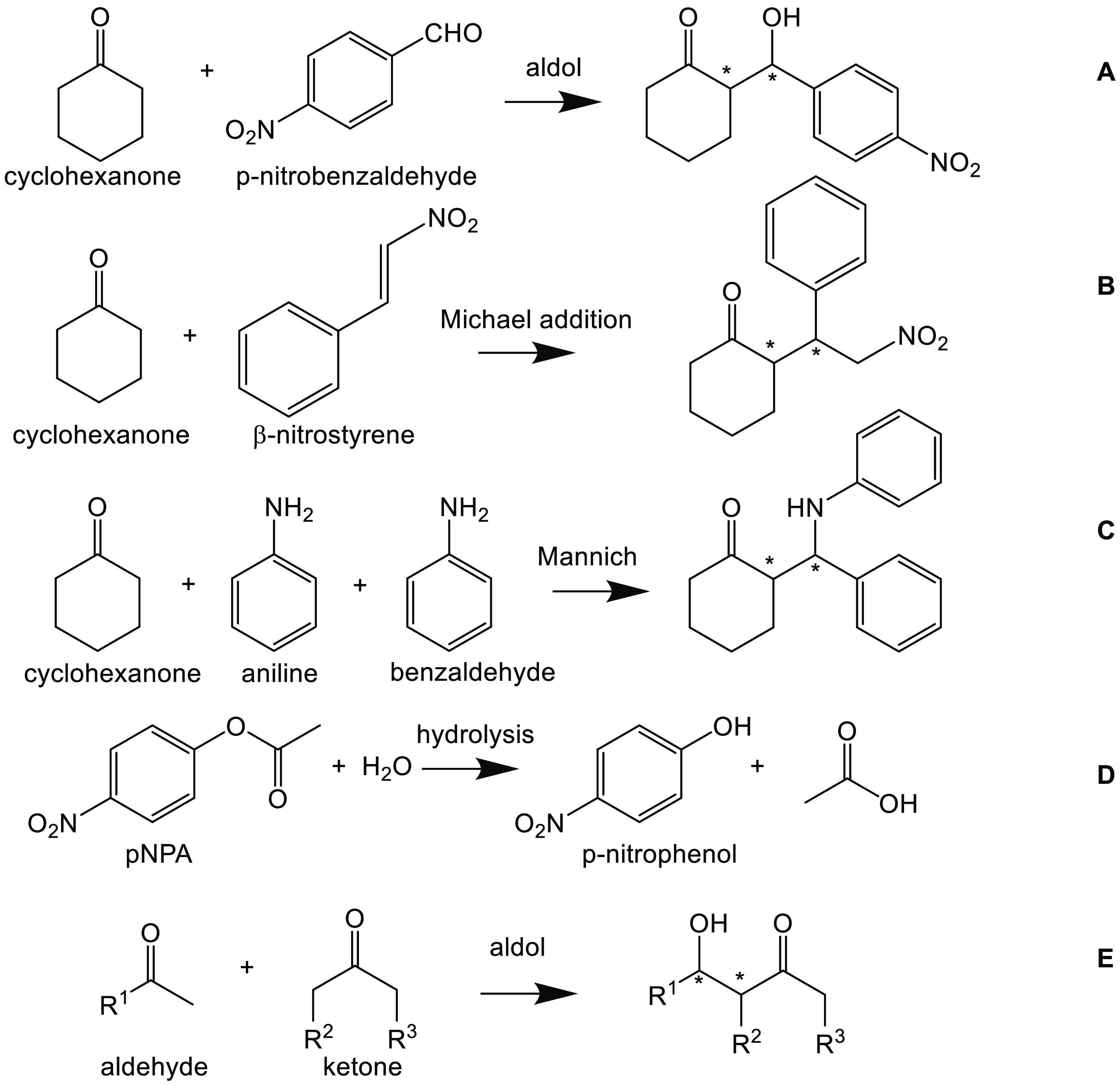
Representative Reactions among Those Discussed Extensively in the Following Text Based on ref (57).

Peptides have been used to catalyze many reactions, as will be evident from the following discussion. Representative reactions that have been the subject of many studies are shown in Scheme 1.

This Review is organized as follows. First, in section 2 the large class of proline-containing peptides and conjugates (and amino acid proline and its conjugates) is considered separately, since in many cases these peptides do not show self-assembly properties. In section 3, examples of research on α-helical peptide assemblies, i.e. coiled coil bundles with catalytic activity, are discussed. In section 4, the catalysis of reactions and the creation of enzyme mimics based on β-sheet peptide fibril and nanotube structures is considered. Section 5 covers peptide catalysts based on the use of peptide supports for metal nanoparticles. Concluding remarks are presented in section 6.

## 2 Self-Assembling Proline-Containing Peptides

There is a large body of research on proline-based peptide catalysts due to the important catalytic role of proline in many types of organic reaction, and many of the proline and proline-based peptides and conjugates have disordered or turn conformations. However, there is overlap of this section with section 4, since some of the proline-based biocatalysts form β-sheet fibrils. In general proline residues disfavor α-helical conformations so there is little overlap with section 3.

List et al. reported in 2000 that l-proline can catalyze an intermolecular aldol reaction.^58^ Since then this amino acid and peptides containing it have been the subject of large numbers of studies of catalytic activity. The activities of proline and derivatives and proline-based peptides in catalyzing a wide range of reactions (cf. Scheme 1), including aldol reactions, acyl transfer, hydrocyanation, Michael addition, and Mannich reactions among many others, have been reviewed.^31,32,57,59−63^ These early studies did not focus on the influence of peptide self-assembly and so are not discussed further here. A wide range of peptides (including some non-assembling peptides not based on proline), reactants, solvent conditions, etc are summarized in these excellent reviews.

Cordova et al. measured the catalytic properties of a wide range of single amino acids (including α-amino acids and β-amino acids) and also chiral primary amines and dipeptides using the model aldol reaction of *p*-nitrobenzaldehyde with cyclohexanone (reaction **A** in Scheme 1).^64^ Among α-amino acids, (*S*)-valine showed a yield for this reaction of 98% and diastereoselectivity of 37:1 *anti*: *syn* and 99% enantiomeric excess. Excellent performance was also observed for several of the dipeptides investigated. Trace water (10 equiv) was found to accelerate the aldol reactions, the performance of which was also compared using different organic solvents. The reactions were also analyzed using a range of ketones in place of cyclohexanone and other aldehyde acceptors in different asymmetric aldol reactions.^64^

**Figure 1:**
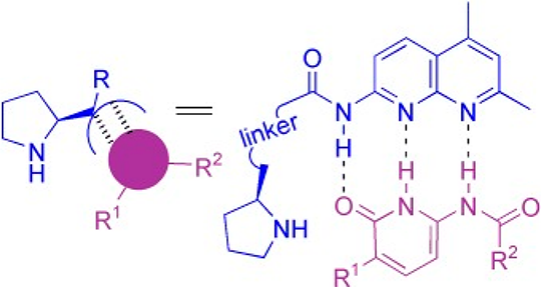
Complexation of proline–naphthyridine with a pyridinone via multiple hydrogen bonds.^65^ Reprinted with permission from ref (65). Copyright 2007 Wiley-VCH GmbH.

In one example of a study on a catalytically active self-assembled proline conjugate, it was shown that proline–naphthyridine peptide conjugates can coassemble via hydrogen bonding with pyridinones (Figure 1), although the self-assemblies do not appear to have ordered structures. The coassembly leads to catalysts that act via enamine intermediates, for example for nitro-Michael addition reactions.^65,66^ The catalytic activity with different pyridinone derivatives and Pro-Nap peptides with different stereochemistries was evaluated.^65^

**Scheme 2:**
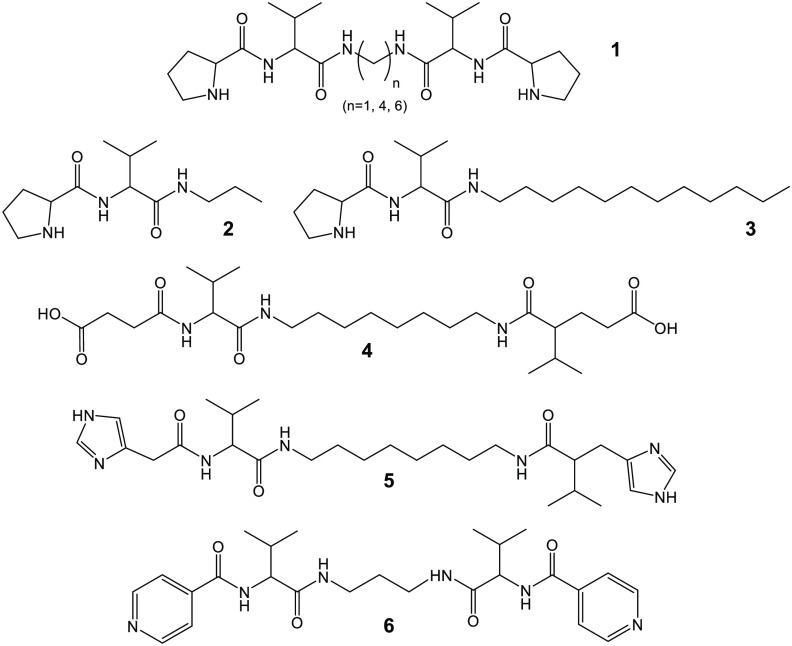
Examples of Peptide-Based Amphiphiles Studied by the Groups of Escuder and Miravet Based on ref (57).

The group of Escuder and Miravet has investigated the catalytic activity of bola-amphiphilic peptides such as **1** in Scheme 2 (for example PV-C_8_-VP).^67,68^ These molecules are organogelators in toluene. Peptide bola-amphiphile PV-C_8_-VP was found to form gels in acetonitrile, with the gels having greatly enhanced activity in model Henry nitro-aldol reactions compared to sols formed above the sol–gel temperature, due to the ordered array of proline residues at the surface of the fibrils that form the gel network structure.^67^ In fact, different products, nitroalkenes, were obtained in solution, via a distinct proposed mechanism. It was shown that the catalyst could be recovered by filtration for reuse.^67^ The activity of the analogue of PV-C_8_-PV with a hexyl spacer in catalysis of the nitro-aldol reaction of *trans*-β-nitrostyrene with cyclohexanone (reaction **B** in Scheme 1) was compared with that of the terminal peptide fragment and the peptide prolinamide analogue.^68^ The bola-amphiphilic gelator shows a higher diastereoselectivity (98:2 *syn*:*anti*) and enantioselectivity (33% e.e. (2R,1′S)).^68^ A lipopeptide analogue (non-bolaamphiphilic PV-C_12_ lipopeptide, **3** in Scheme 2) is a hydrogelator that shows high stereoselectivity and yield for the *p*-nitrobenzaldehyde/cyclohexanone nitro-aldol reaction (reaction **A** in Scheme 1).^69^ The catalyst system also shows a useful property of recyclability after breakup of the gel (in response to mechanical deformation and/or temperature), with the gel reforming in response to pH or temperature. This lipopeptide also shows different fibrillar polymorphs depending on the sample preparation process (temperature, aging time, pH, use of ultrasound).^70^ The same group also compared PX-C_4_ (X = V, I, F, A) lipopeptides as catalysts for the conjugated addition of cyclohexanone to *trans*-β-nitrostyrene in toluene (reaction **B** in Scheme 1).^71^ Although aggregation of the derivatives was noted (as determined from NMR experiments of the concentration dependence of amide resonances), the nature of the self-assembly in the organic solvent was not examined, although it was reported that the catalyst is activated by self-aggregation. Furthermore, conformational differences were expected (based on molecular modeling) comparing the derivatives. The F and A derivatives show a lower degree of aggregation and catalytic performance in terms of enantioselectivity, ascribed to the presence of a transition from *anti* to *syn* conformation upon aggregation.^71^ As well as organogelators, this group also showed that lipopeptides such as PV-C_12_ (**3** in Scheme 2) form catalytically active hydrogels, for example for direct aldol reactions of aliphatic ketones of varying chain length with 4-nitrobenzaldehyde, with the yield significantly increasing with the hydrophobicity of the ketone.^72^ In another example, hydrogels based on β-sheet fibrils are formed by PFE-C_12_ and PEF-C_12_ (and related sequences), and these also show catalytic properties.^73^ Examining a model aldol reaction, the authors found that aggregated peptides are catalytically active, in contrast to nonaggregated catalytically inactive analogues. Furthermore, attachment of lipid chains provided a hydrophobic environment that improves solubilization of hydrophobic moieties and hence catalytic performance. As a model for a biomimetic aldolase, the lipopeptides were also used in catalytic studies of the self-condensation of several oxyaldehydes and phenylalkylaldehydes, which revealed good yield and stereoselectivity for some substrates.^73^

In a development of this work, the structure and rheological properties of related organogelators (molecules **1** in Scheme 2) in acetonitrile or toluene were examined, including the orientation of the β-strands (deduced from FTIR experiments) in the fibrillar network which was imaged by SEM and cryo-SEM while the macroscopic superstructure was resolved using optical microscopy.^74^

This group also showed that their PV lipopeptides are able to catalyze other reactions, including 1,4-conjugated addition of ketones and alkenes (e.g., cyclohexanone and *trans*-β-nitrostyrene, reaction **B** in Scheme 1)^68^ and an antiselective Mannich reaction.^75^ In the latter case PV lipopeptides were supplemented with carboxylic acid functionalized conjugates (SucVal-C_8_-ValSuc bola-amphiphile, Suc: succinate, **4** in Scheme 2), enabling three-component Mannich reactions of cyclohexanone, aniline, and benzaldehyde (reaction **C** in Scheme 1) in the sol–gel phase (sol of SucVal-C_8_-ValSuc) with gel fibers of the PV-C_12_ lipopeptide.^75^ However, when SucVal-C_8_-ValSuc was coassembled with a structurally similar catalytically active hydrogelator (ProVal-C_8_-ValPro), the resulting coassembly did not improve selectivity and efficiency.^75^ The same conslusion applied to one-pot deacetalization–aldol tandem reactions, i.e. when the same two peptide bola-amphiphiles were coassembled, self-sorting was precluded and no tandem catalysis was observed.^76^

Lipopeptide PW-C_12_ and analogues have been shown to catalyze the aldol reaction of cyclohexanone with nitrobenzaldehyde in water.^77^ The mode of self-assembly in water changes from spherical nanostructures to fibrillar gels, depending on the nature of the cosolvent. The enantiomeric selectivity of the aldol reaction catalyzed by nanofiber gels was found to be much lower than that achieved with nanosphere structures, pointing to the role of the shape of the self-assembled structure on access to the catalytic site.^77^ With use of compressed CO_2_, it is possible to drive the formation of vesicle-like structures by PW-C_12_, and the yield and enantiometric excess can be tuned depending on CO_2_ pressure, which influences vesicle size.^78^

**Figure 2:**
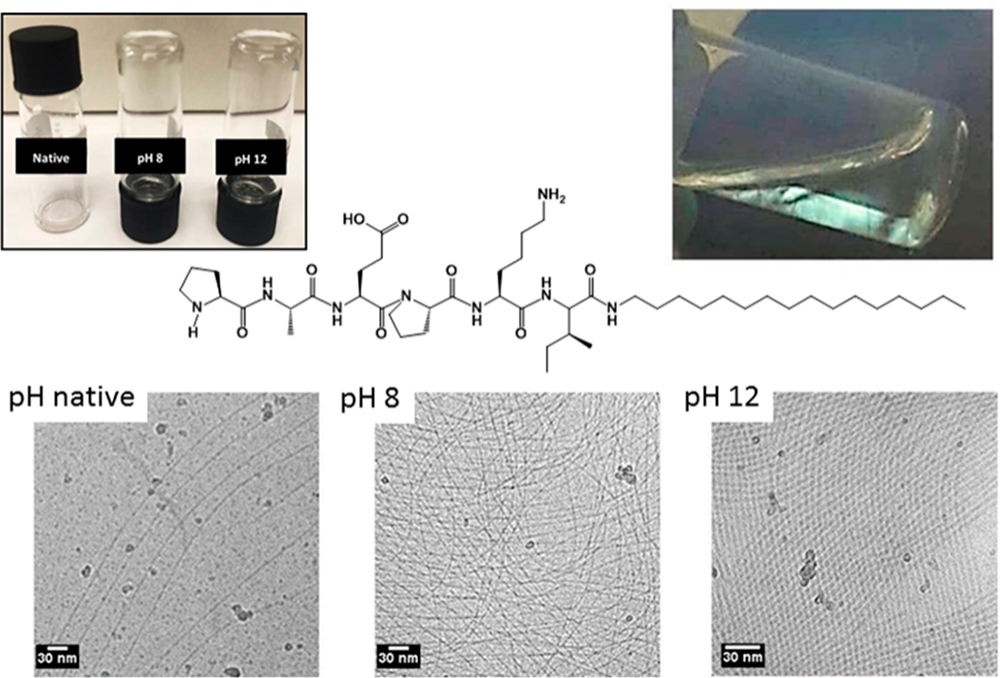
Fibrils formed by catalytically active lipopeptide PAEPKI-C_16_ shown in the center.^83^ TEM images at three pH values (bottom) along with images of the lipopeptide in aqueous solution at the three pH values indicated, showing formation of gel at high pH (top left) and an image of a solution at higher concentration imaged between crossed polarizers showing birefringence due to nematic ordering of the fibrils at pH 8 (top right). Reprinted with permission from ref (83). Copyright 2020 American Chemical Society.

Lipidated proline derivatives have been investigated as catalysts for several reactions. Hayashi et al. demonstrated the high enantioselectivity and disastereoselectivity that can be achieved in aldol reactions using a range of lipidated proline derivatives (C-terminal proline with C_6_–C_16_ chains) as well as proline itself and other derivatives, although they did not study self-assembly behavior.^79^ In fact, lipidated proline peptides (C_8_, C_12_, C_16_ chains, C-terminal proline) show interfacial activity in the stabilization of oil–water emulsions such as those formed by water and cyclohexanone, which are used in model aldol reaction catalysis studies.^80^ In this sense, the amphiphilicity of such molecules can influence their assembly, and the properties of the solution phase and hence catalysis and emulsion formation should be examined in this context. In another study, it was shown that lipidated proline (ether-linked C_12_ and C_6_ alkyl chains with C-terminal proline) conjugates have high yield and excellent enantioselectivity and diastereoselectivity in direct aldol reactions.^81^ Although the crystal structure of one of the lipopeptides was reported, self-assembly properties were not examined in this work. The location of the proline residue and lipid chain can have a profound effect on the self-assembly behavior and catalytic properties, as exemplified by a study that compares C_16_-IKPEAP with PAEPKI-C_16_ (reverse sequence with free N-terminal proline). The former lipopeptide self-assembles into spherical micelles in aqueous solution (with a disordered peptide conformation),^82^ but the latter forms β-sheet fibrils over a wide range of pH (Figure 2).^83^ In a study of a model nitro-aldol reaction (reaction **A** from Scheme 1), PAEPKI-C_16_ shows enhanced *anti*:*syn* diastereoselectivity and better conversion compared to C_16_-IKPEAP.^83^ In another study, the nature of the linker between the peptide and the lipid chain was examined. While it did not influence the self-assembly of lipopeptides PRW-NH-C_16_ (amide linker) or PRW-O-C_16_ (ester linker) into spherical micelles, the former does show enhanced catalytic activity for a model nitro-aldol reaction, this being ascribed to differences in the local conformation around the catalytic site and/or the altered polarization of the amide vs ester linkage.^84^ These lipopeptides contain a tripeptide sequence with free N-terminal proline for catalytic activity, an arginine residue to improve solubility, and a tryptophan residue for fluorescence detection.^85^ The amide linked peptide is also expected to show greater stability, in particular being resistant to ester hydrolysis.

A Brazilian group has very recently developed a range of proline-based amyloid peptides and lipopeptides which form self-assembled micelles, nanotape, and fibril structures and show excellent catalytic activity and high selectivity for aldol reactions in aqueous solution.^83,86,87^ The group investigated the self-assembly and catalytic activity of mixtures of PRWG-C_18_ which contains a free N-terminal proline catalytic residue with noncatalytic homologue RWG-C_18_ (as diluent and to facilitate self-assembly). The conversion for the asymmetric aldol reactions using cyclohexanone and *p*-nitrobenzaldehyde could be optimized to exceed 94% with a very high enantioselectivity, 93:7 *anti*:*syn*.^86^ In water, mixtures of the lipopeptides were found to self-assemble into nanotapes based on a bilayer structure with the exception of mixtures rich in PRWG-C_18_ which form micelles. The self-assembly of mixtures of lipopeptides with two lipid chains, PRWG-(C_18_)_2_ and RWG-(C_18_)_2_, was also examined, and these also form a nanotape structure in water. In water/cyclohexanone mixtures, the lipopeptide mixtures (of either single chain or double chain lipopeptides) formed fractal aggregate structures, on the basis of form factor fitting of measured SAXS data.^86^ This group also investigated the catalytic performance of peptides [RF]_4_ and P[RF]_4_ in a model nitro-aldol reaction (reaction **A** from Scheme 1).^88^ These peptides form either fibrils or globular structures depending on pH and concentration. Unexpectedly, the diastereomeric ratio and enantiomeric excess were higher for the former non-proline functionalized peptide which was ascribed to a more compact active site structure for the former peptide in conditions where it forms globular aggregates.^88^

Among other examples, proline-functionalized lipopeptides (even as short as lipidated dipeptides) which form vesicles promote transfer hydrogenation of ketones in the aqueous phase with excellent conversion rates and enantioselectivities (>90% ee).^89^ Lipidated proline derivatives can successfully catalyze asymmetric aldol reactions, with the conversion and stereoselectivity depending on the self-assembled structure in solution.^74,90−95^ Changes in peptide sequence lead to new modes of self-assembly, through a combination of different supramolecular interactions, such as π-stacking and electrostatic and hydrogen bonding,^85,88^ and this in turn can have a profound effect on catalytic activity.

In another example of a catalytically active self-assembling proline peptide conjugate, nanotubes are formed in aqueous solutions of a PK peptide with 1,4,5,8-naphthalenetetracarboxylic acid diimide (NDI) attached at the ε-amino group of the lysine residue.^96^ The peptide nanotubes are able to catalyze nitro-aldol reactions, good diastereomer excess and enatiomeric excess being achievable. The nanotubes can be recovered by ultracentrifugation, providing a method to recycle the catalyst.^96^ Other examples of catalytically active peptide nanotube structures are discussed in section 4.

A lipopeptide with a short β-hairpin sequence (^D^Pro-Gly turn) attached to a myristyl chain is able to bind hemin and thus act as a peroxidase in a micellar surfactant solution.^97^ The myristyl chain is incorporated to facilitate binding to dodecylphosphocholine (DPC), and the oxidation of tetramethylbenzidine (TMB) in the presence of hydrogen peroxide in the micellar solution was quantified.

## 3 Catalysts Based on Helical Constructs

First, we note that the creation of catalytic pockets based on the design of α-helical folds in peptides and proteins has been the subject of numerous studies and previous reviews.^13,14,19,37,38,57,98^ Here, as mentioned above, we do not consider designed catalytic protein structures or natural enzymes, and the following section highlights examples of studies on catalytic α-helical peptides, usually those with a coiled coil structure.

In an earlier study, Benner et al. created two 14-residue α-helical oxaloacetate decarboxylases (termed Oxaldie 1 and 2) by consideration of the mechanism of the decarboxylation reaction which occurs via an imine intermediate.^99^ This led to the design of a helix with a lysine-rich hydrophilic face and a leucine/alanine-rich hydrophobic face.

Allemann’s group later developed Oxaldie 3, a 31-residue peptide designed to function as an oxaloacetate decarboxylase, based on a helical peptide sequence from a pancreatic peptide (PP) with three lysine substitutions.^100^ The performance of the peptide in the catalysis of the decarboxylation of oxaloacetate to produce pyruvate was analyzed by following the conversion of NADH to NAD [NAD: nicotinamide adenine dinucleotide]. The design of the peptide was later significantly improved in Oxaldie 4, based on bovine pancreatic peptide (bPP) which showed a tightly packed structure comprising a poly proline-like helix and an α-helix, in sharp contrast to the molten globule-like structure formed by Oxaldie-3 (deduced from ^1^H NMR spectra), which was based on avian pancreatic polypeptide (aPP).^101^ The stability of Oxaldie-4 with respect to thermal and urea denaturation was also significantly improved in comparison to Oxaldie-3. Despite this, the catalytic activity was not greatly improved, this being ascribed to the flexibility of the lysine residues which form the active site^101^ (with a significantly lower p*K*_a_ than the usual value for lysine due to the presence of a second nearby amino group^100^). Later, this group prepared a related 31-residue bPP-based peptide ArtEst, also expected to form an antiparallel helix–loop–helix structure comprising an N-terminal polyproline type-II helix that is connected to the C-terminal α-helix by a type II β-turn.^102^ Pancreatic polypeptides form stable dimers over wide ranges of concentration and pH, and NMR provided evidence for a parallel dimer structure of ArtEst. The bPP peptide was modified by removal of C-terminal residues not involved in the fold but with substitutions of histidine residues to confer catalytic esterase activity.^102^ The performance of this in ester hydrolysis catalysis is compared to other peptides discussed in this review in Table 1.

**Table 1:** Hydrolase Activity Reported for Peptides (and Carbonic Anhydrase), for Reaction **D**, with One Exception for an Analogue of *p*-Nitrophenyl Acetate (pNPA)^a^

Peptide/amino acid	Substrate	*k*_cat_/*K*_M_ (M^–1^ s^–1^)	*k*_cat_/*K*_M_ × 10^–2^ ((gl^–1^)^−1^ s^–1^)	Reference
**β-sheet-based peptides**				
Ac-IHIHIYI-NH_2_^b^	pNPA	355 (pH 8.0)	37	(105)
Ac-IHIHIQI-NH_2_^b^	pNPA	62 (pH 8.0), 15.76 (pH 7.3)	6.7 (pH 8.0), 1.7 (pH 7.3)	(106, 107)
Ac-YVHVHVSV-NH_2_^b^	pNPA	6.29 (pH 7.3)	0.64	(107)
HK_H_-LLLAAA(K)-palmitoyl^b^^,^^c^	DNPA^d^	19.76 (pH 7.4)	1.4	(108)
C_12_-VVAGH + C_12_-VVAGS + C_12_-VVAGD	pNPA	126.6 (pH 7.5)	19.8	(109)
HSGVKVKVKVKV^D^PPTKVKVKVKV-NH_2_^b^	pNPA	19.18 (pH 9.0)	0.76	(110)
Fmoc-FFH-NH_2_ + Fmoc-FFR-NH_2_^b^	pNPA	1.82 (pH 7.5)	0.13	(111)
HSGQQKFQFQFEQQ-NH_2_ + RSGQQKFQFQFEQQ-NH_2_^b^	pNPA	0.15 (pH 7.5)	4.2 × 10^–5^	(112)
HV-C_8_-VH	pNPA	5.3 (pH 7.0)	0.94	(113)
F^b^	pNPA	76.54 (in H_2_O), 10.62 in Tris-HCl solution (both pH 7.0)	46 (in H_2_O), 6.4 (in Tris-HCl buffer)	(104)
**α-helical peptides**				
Art-Est	pNPA	0.014 (pH 5.1)	3.8 × 10^–4^	(102)
MID1-zinc^b^	pNPA	35 (pH 7.0)^e^	0.65	(114)
(TRIL9CL23H)_3_^b^	pNPA	1.38 (pH 7.5)	0.040	(115)
Alleycat E	pNPA	5.5 (pH 7.5)	0.064	(116)
Alleycat E2	pNPA	6 (pH 7.5)	0.069	(116)
CC-Hept-CHE	pNPA	3.7 (pH 7.0)	0.11	(103)
CC-Hept-(hC)HE (hC: homocysteine)	pNPA	1.9 (pH 7.0)	0.056	(103)
SHELKLKLKL + WLKLKLKL conjugated to carbon nanotubes	pNPA	0.62 (pH 7.5)		(117)
S-824	pNPA	6.25 (pH 8.5)	0.05	(118)
**Native Enzyme**				
Carbonic anhydrase^b^	pNPA	1670 (pH 7.0)	5.7	(119)

a Adapted from refs (103 and 104).

b With Zn^2+^.

c K_H_ denotes a histidine attached via lysine ε-amino (Scheme 3), with the palmitoyl chain also being attached via a lysine linker.

d 2,4-Dinitrophenylacetate.

e Significantly higher at higher pH.

**Figure 3:**
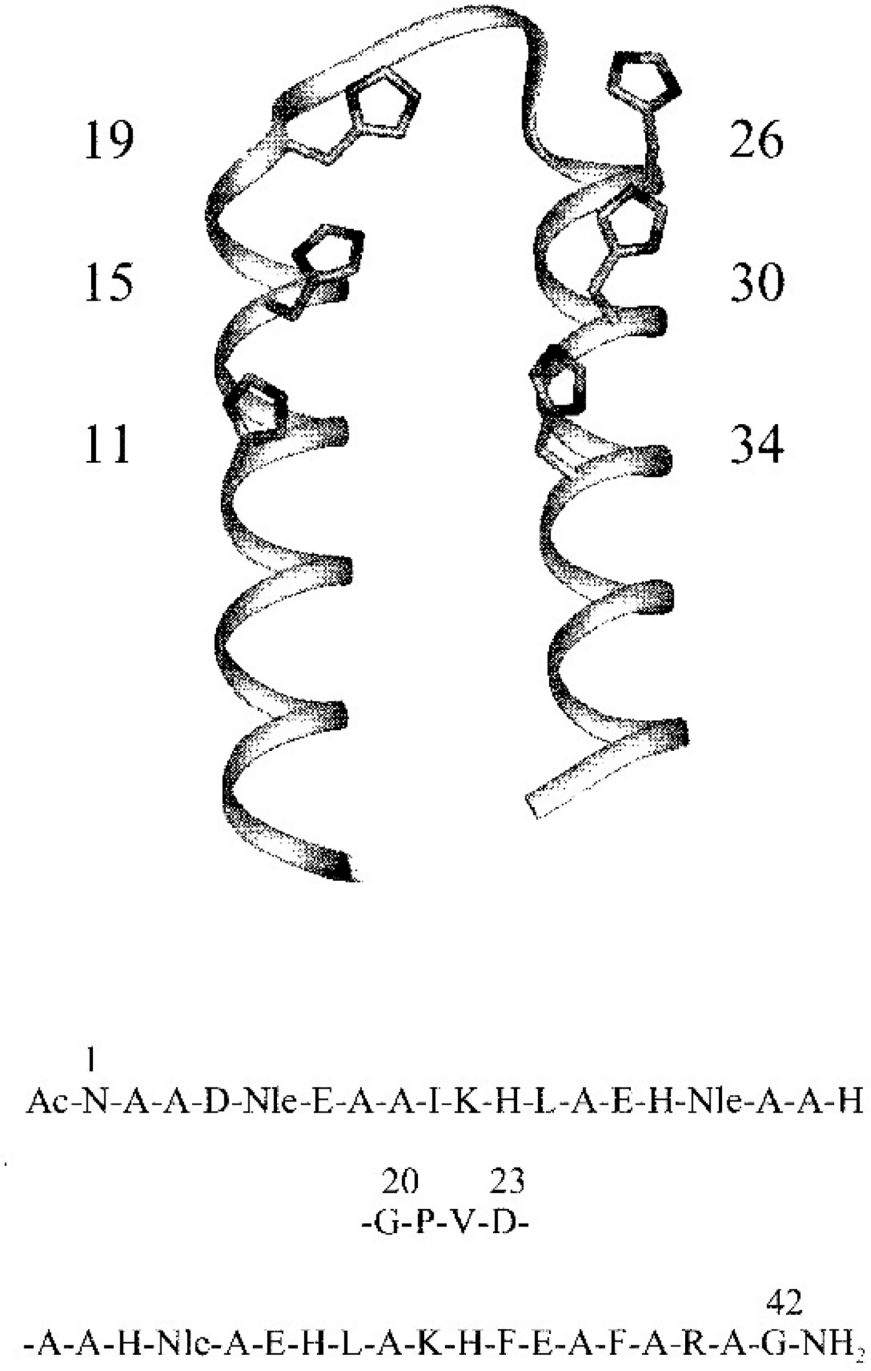
Secondary structure representation (showing catalytic histidine residues) and sequence of catalytically active helix–loop–helix peptide KO-42 with the loop region GPVD shown in the middle of the sequence at the bottom.^120^ Reprinted with permission from ref (120). Copyright 1998 American Chemical Society.

Baltzer’s group developed a 42-residue peptide (KO-42, Figure 3) designed with six histidine residues to form a catalytically active helix–loop–helix structure that forms a dimer, the structure of which was elucidated using CD, NMR, and ultracentrifugation.^121^ The four-helix bundle structure was able to catalyze hydrolysis (reaction **D** from Scheme 1) and transesterification reactions of *p*-nitrophenyl esters. The p*K*_a_ values of the histidine residues were grouped around p*K*_a_ = 5 or p*K*_a_ = 7, producing an active site containing both protonated and unprotonated residues.^121^ In subsequent work to elucidate the mechanism of catalysis, this group prepared variants of the peptides with histidine substitutions and/or additional arginine residues in the helix adjacent to the helix bearing the histidine catalytic site.^120,122^ These studies highlighted the importance of the histidine p*K*_a_ values (appropriate values of which lead to the presence of catalytically active HisH^+^–His pairs) on the catalytic activity, as well as the role of arginine in improving binding in the transition state.^120^ The group also improved the function of the HisH^+^-His site by examining the influence of flanking R and K residues to provide recognition of substrate carboxylate and hydrophobic residues. This led to chiral recognition, demonstrated by the fact that the hydrolysis of the *p*-nitrophenyl ester of d-norleucine was catalyzed with a second-order rate constant twice that of the l-norleucine analogue.^122^ Baltzer et al. also developed their peptide design for the catalysis of other reactions. They screened variants based on the same 42-residue helix–loop–helix peptide to catalyze the transamination reaction of aspartic acid to form oxaloacetate,^123^ and selected lead candidates were screened for their ability to bind aldimine intermediates. Capping residues and charged residues capable of salt bridge formation and of helical dipole stabilization were also introduced to increase the stability of the helices.^123^ The same peptide scaffold was used to catalyze hydrolysis of a more challenging uridine (phosphodiester) substrate as a model for an RNA cleavage reaction, using a designed peptide with additional arginine residues substituted (among other charged residues).^124^ The second order rate constant was increased 250 times compared to the reaction catalyzed only by imidazole groups. The catalytic activity was further enhanced by incorporation of tyrosine close to the active site based on two histidine residues flanked by four arginines and two adjacent tyrosine residues.^125^

DeGrado’s group developed a heterotetrameric peptide that self-assembles from three types of subunits (two “A” chains and one “B” chain, all containing 33-residue designed sequences).^126^ The peptide forms a complex with iron ions in an asymmetric helical bundle. This noncovalent self-assembling helical peptide complex with iron was shown to be an efficient O_2_-dependent phenol oxidase, able to catalyze the two-electron oxidation of 4-aminophenol.^127^ It was also shown the activity is specific, since glycine substitutions significantly affected the activity. This group also developed a 114-residue peptide that forms a four-helix helical bundle that can bind a range of divalent metal ions, including iron.^128^ This peptide was used as the basis to design variants able to catalyze O_2_-dependent, two-electron oxidation of hydroquinones or selective *N*-hydroxylation of arylamines.^129^

**Figure 4:**
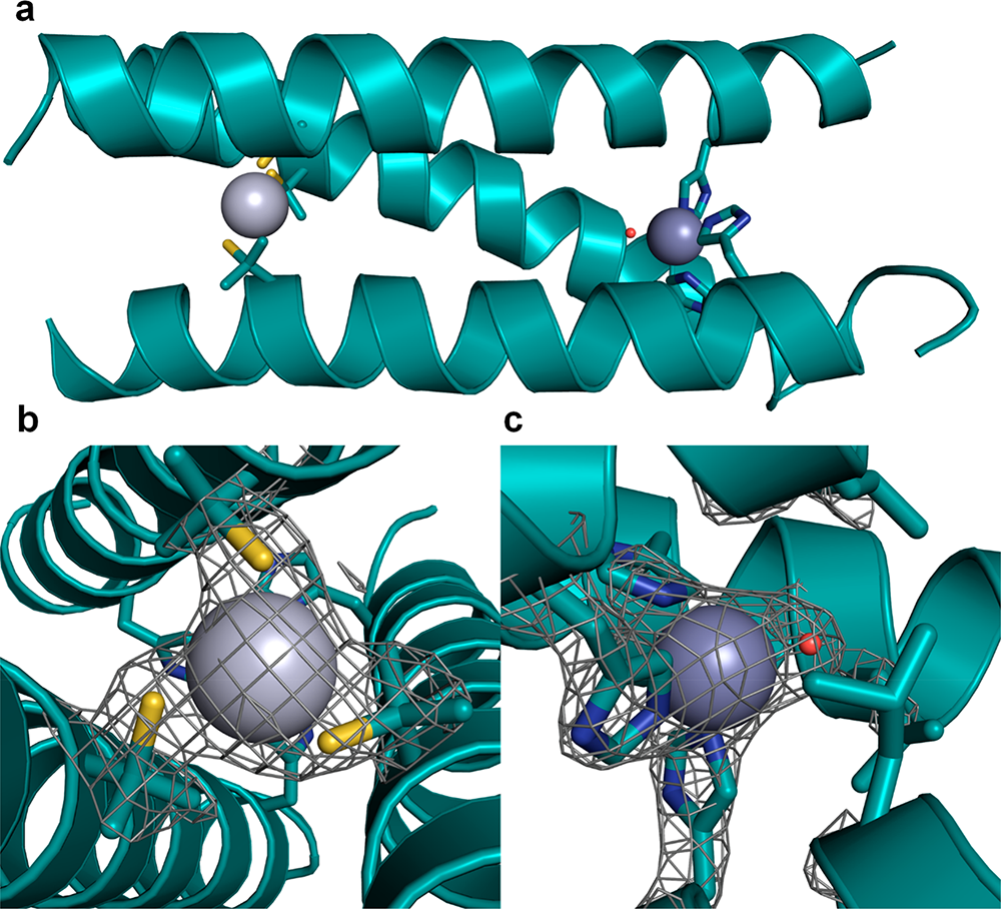
Structure of a designed metalloprotease three-helix bundle [pdb 3PBJ]. (a) One trimer with bound metal ions. (b) Enlargement of site showing C residues around a Hg^2+^ ion (with electron density grid overlaid). (c) H residues around a Zn^2+^ ion. Reprinted by permission from Springer-Nature: Nature Chemistry ref (115). Copyright 1998.

Three-helix bundles which can bind heavy metal ions in a tricysteine domain (Figure 4) and which were designed to serve as model metalloenzymes have been developed by Pecoraro’s group^130^ based on a design by DeGrado’s group.^131^ The catalytic esterase activity has been measured for one of these peptides, termed TRIL9CL23H. The molecular structure (based on single crystal XRD) from a variant of this peptide is illustrated in Figure 4,^115^ revealing a Hg^2+^ ion bound by a tricysteine motif as well as a histidine Zn^2+^-binding region (Figure 4), with the latter resembling those observed for carbonic anhydrase and matrix metalloproteases. The peptides, based on a modification of a (LKALEEK)_4_ sequence to incorporate histidine and/or cysteine (or penicillamine) residues, bind to Zn^2+^ for catalytic activity. Binding of Hg^2+^ with cysteines present in some peptides examined provides structural stability. The activity was assayed using pNPA hydrolysis, and values at pH 7.5 are listed in Table 1.^115^ The effect of the location of the active site (Hg(II)-bound tris-thiolate site and the Zn(II)(His)_3_ solvated site) was later explored in a number of variants of the 3-helix bundle peptide, and it was shown that activity for pNPA hydrolysis was retained, although substrate access, maximum rate, and metal binding affinity changed.^132^ For all variants studied, the catalytic efficiency is reported to increase with pH up to the highest pH ∼ 9.5 examined.^132^ The same peptide scaffold can bind copper ions, and this was used to produce Cu(I/II)(TRIL23H)_3_ which lacks the cysteine residues (of the Hg^2+^ and other heavy metal ion binding peptides) and has sequence Ac-G[LKALEEK]_3_[HKALEEK]G-NH_2_. This peptide acts as a functional model for the active site of copper nitrite reductase.^133,134^ The structure and catalytic activities of the TRI series have been reviewed.^135,136^

In addition to this parallel 3-helix bundle peptide, the Pecoraro group also developed peptide metalloenzymes based on an antiparallel 73-residue (single chain) 3-helical bundle protein developed in the DeGrado lab, termed α_3_D.^137−140^ Substitution of three leucine residues to histidines (and H72V substitution, also a small peptide extension) led to peptide α_3_DH which is effective in the hydration of CO_2_, being within an order of magnitude of the efficiency of one form of carbonic anhydrase.^141^

Degrado’s group recently showed that three-helix bundles formed by domain-swapped 48-residue dimers (DSDs) can be designed as catalysts containing oxyanion-binding sites which were active in acyl transfer reactions such as peptide ligation through transthioesterification and aminolysis.^142^ The heteromeric dimers contain pairs of peptides bearing complementary E or R residues, the anionic peptide being functionalized with an active cysteine residue near its N-terminus to facilitate the reaction with a peptide-^α^thioester substrate.

**Figure 5:**
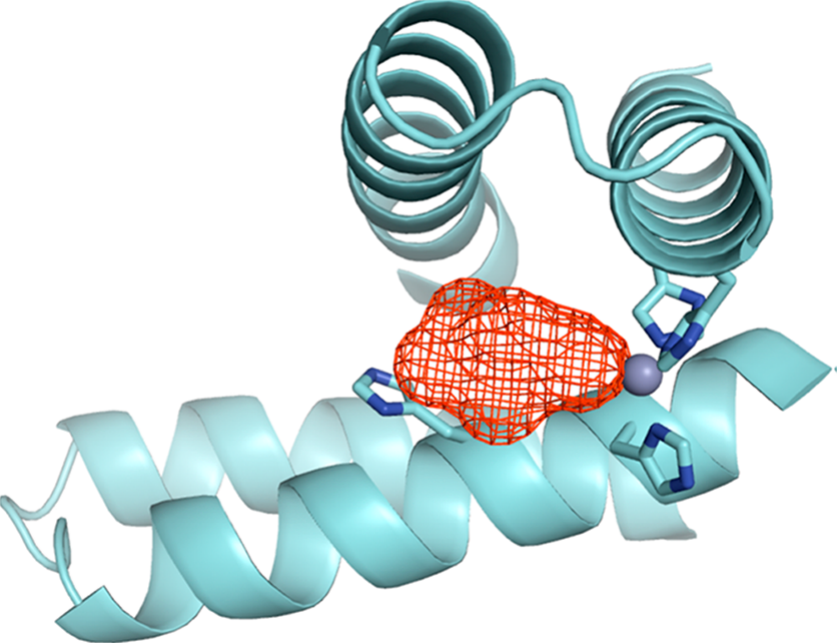
Homodimeric coiled coil based on a de novo design, showing cleft (red mesh) and Zn^2+^ (sphere) binding site with highlighted histidines in the catalytic site.^114^ Reprinted with permission from ref (114). Copyright 2012 American Chemical Society.

A homodimeric coiled coil (based on a 46-residue helix-turn-helix, i.e. hairpin-shaped, monomer) was designed de novo^143^ to feature a Zn^2+^ binding domain to facilitate catalytic hydrolysis.^114^ The peptide, termed MID1-zinc for zinc-mediated homodimer, is illustrated in Figure 5. This peptide has high catalytic activity for pNPA hydrolysis (Table 1), especially at high pH, where *k*_cat_/*K*_M_ = 660 M^–1^ s^–1^ at pH 9 for example.^114^

**Figure 6:**
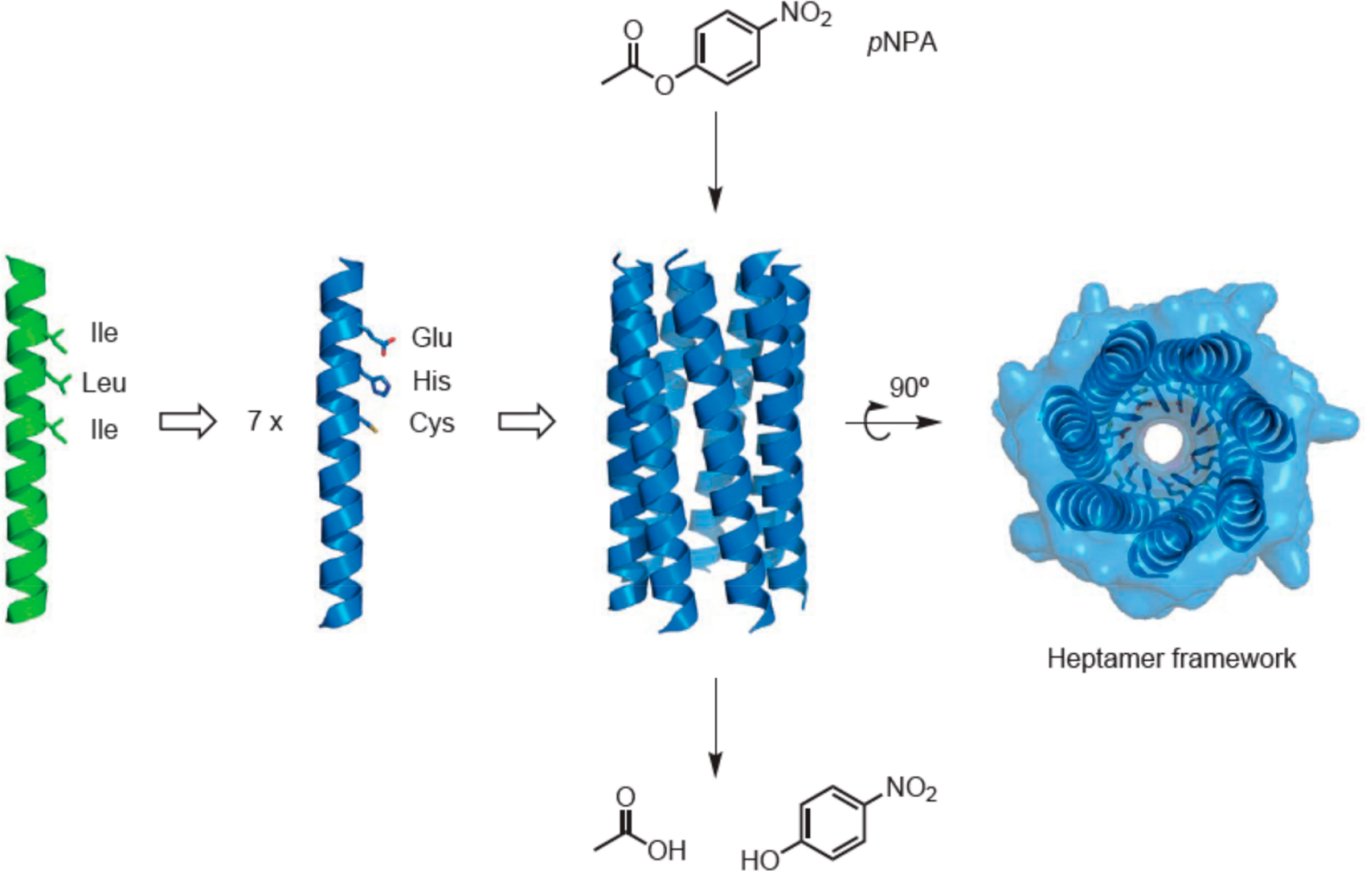
Heptameric coiled coil designed by Woolfson’s group which acts to hydrolyze pNPA. The parent peptide sequence (green) was substituted with the E, H, C triad in the catalytically active heptameric structure (blue). From ref (144) based on ref (103). Reprinted by permission from Springer-Nature: Nature Chemistry ref (144). Copyright 2016.

Woolfson’s group have developed homoheptameric coiled coil structures that function as esterases.^103^ These structures contain a hydrophobic pore that facilitates access of the substrate (in this case pNPA, Figure 6). Inspired by natural hydrolases, the peptides were designed to contain a catalytic triad consisting of a glutamate, a histidine, and a cysteine.^103^

A heterotetrameric coiled coil has been designed to covalently bind heme (by incorporating a *bis*-histidine motif) and was shown to act as a thermostable artificial enzyme that catalyzes a diverse array of substrate oxidations coupled to the reduction of H_2_O_2_, similar to those of natural heme-containing peroxidases.^145^ The design was inspired by the concept of a protein/peptide “maquette”^146−148^ for de novo c-type cytochromes with enhanced structural stability,^149,150^ which undergo post-translational modification in *E. coli* to covalently graft heme onto the protein backbone.

A partially α-helical peptide (bPP) has been used as a template to design catalysts for Diels–Alder and Michael addition reactions.^151^ The peptide also has a polyproline type II helix domain (residues 1–8) and a turn (9–12), with the α helix comprising residues 13–31. Substitutions in the sequence were made to incorporate histidine or (3- or 4-)pyridylalanine in order to bind Cu^2+^ within the “metallopeptide enzyme”. Good enantioselectivities were reported, along with high substrate selectivities.

Hecht’s group has designed de novo coiled-coil tetramers (four-helix bundles) active as hydrolases^118^ peroxidases and others^152^ or ATPases.^153^ They developed peptides through combinatorial screening based on the patterning of hydrophobic residues, since positioning a nonpolar residue every third or fourth residue favors α-helical ordering, whereas alternating polar and nonpolar residues promotes β-sheet formation.^154^ The former design led to a 102-residue peptide termed S-824, expressed in *E. coli*, that forms a four-helix bundle. This is able to catalyze the hydrolysis of pNPA (Table 1).^118^ A “superfamily” of ∼10^6^ 102-residue 4-helix bundles was then prepared by expression in *E. coli*, and heme binding was assessed along with activity in model peroxidase, esterase, and lipase reactions.^152^ Catalytic activities well above baseline were observed, although they were generally lower than those of natural enzymes. In a development of this work, the group screened >1100 novel sequences for the ability to hydrolyze either pNP-palmitate or pNP-phosphate, and the lead candidate 101-residue four-helix bundle peptide from this screening process was then studied as an ATPase. Unlike natural ATPases, the activity of the enzyme mimic could be blocked using magnesium ions.^153^

## 4 Catalysts Based on β-Sheet Structures

**Figure 7:**
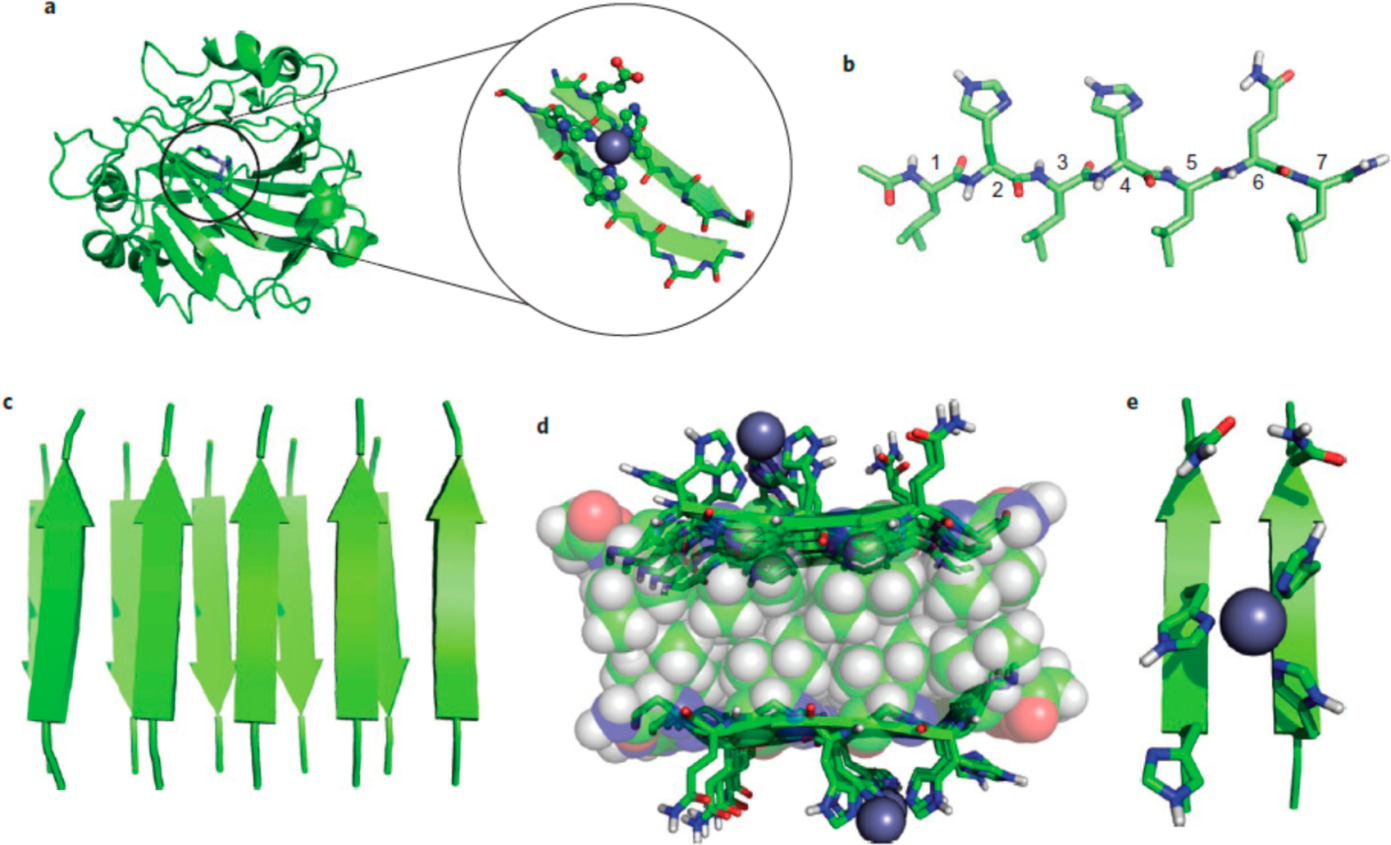
Design of a Zn^2+^-dependent esterase. (a) Structure of human carbonic acid, showing expansion of the Zn^2+^ binding motif. (b) Peptide Ac-LHLHLQL-NH_2_ with residues labeled. (c–e) Computational model of β-strand packing of this peptide showing (c) the overall structure of the array which constitutes a fold mimic, (d) hydrophobic core packing, and (e) the zinc coordination sphere. Reprinted from Springer-Nature: Nature Chemistry, ref (106), Copyright 2004.

Simple short designed amyloid peptides can function as effective catalysts, for example as Zn^2+^-dependent esterases.^38,104,106,155^ Short amyloid peptides have been used as model metalloproteases. The concept is illustrated in Figure 7. A typical metal binding motif based on β-sheets is shown for human carbonic anhydrase in Figure 7a. A series of peptides were prepared based on the alternating “minimal” β-sheet sequence LKLKLKL with alternating hydrophobic and charged residues (as discussed in the preceding section). The lysine residues were substituted at positions 2 and 4 with histidine, capable of binding Zn^2+^ and thus enabling catalysis, while the effect of acidic, basic, or neutral residues at position 6 was examined. Figure 7b shows a representative peptide. Figure 7c–e shows a model for the β-strands which adopt an antiparallel array in the “fold” along with the location of the Zn^2+^ ions. These serve as a cofactor in the catalysis process, and they aid the stabilization of the β-sheet structure. Using pNPA as substrate for the ester hydrolysis (reaction **D** from Scheme 1), a Michaelis–Menten catalytic efficiency up to *k*_cat_/*K*_M_ = 30 ± 3 M^–1^ s^–1^ was reported for the peptide Ac-LHLHLQL-NH_2_ (Figure 7b) which is better on a per weight basis than that of carbonic anhydrase.^106^

The structure of the fibrils formed by active and inactive versions of peptides from this study was later determined by solid state NMR^155,156^ and computer modeling,^155^ which provided information on the relationship between the parallel or antiparallel β-sheet structure and the catalytic activity. These structural studies revealed that the high-activity Ac-IHIHIQI-NH_2_ peptide and its analogue, with Q6Y substitution which shows even higher activity, adopt a twisted parallel β-sheet structure while the lower activity peptide Ac-IHIHIRI-NH_2_ forms a planar antiparallel β-sheet assembly.^155^ In another study, fiber XRD was used to probe the fibril structure of the Ac-IHIHIYI-NH_2_ peptide and related derivatives (in the presence and absence of Zn^2+^), for which the Zn^2+^-dependent pNPA hydrolysis was also assayed.^105^ Riek and co-workers examined the catalytic activity of a series of related designed amyloid peptides with alternating charged/uncharged residues with general sequence Ac-YV**X**_**1**_V**X**_**2**_V**X**_**3**_V-CONH_2_ with **X**_**1**_, **X**_**2**_, **X**_**3**_ = A, D, H or S (as well as Ac-IHIHIQI-NH_2_, for comparison), the catalytic activity for the most active example being shown in Table 1.^107^ It has been shown that the catalytic yield of this peptide (Ac-YVHVHVSV-NH_2_) and Ac-LHLHLRL-NH_2_ can be enhanced by application of pressure with a high-pressure stopped flow system, as exemplified by pNPA hydrolysis studies for which the catalytic efficiency increased to *k*_cat_/*K*_M_ = 450 M^–1^ s^–1^ for Ac-IHIHIQI-NH_2_ at 200 MPa and 38 °C.^157^

Fibril forming Ac-IHIHIQI-NH_2_ is able to catalyze reactions other than hydrolysis. For example it was examined as a catalyst (with Zn^2+^ cofactor) for a number of ester hydrolysis reactions using N-terminal Boc or *N*-benzyloxycarbonyl (Z) protected l-phenylalanine or l-asparagine *p*-nitrophenyl esters.^158^ The catalytic activity (and enantiomeric selectivity) was significantly higher comparing the more hydrophobic l-Phe Z derivative to the Boc analogue, and the authors thus highlighted the importance of hydrophobic interactions between the catalyst and the substrate. The catalysis using peptides undergoing fibril formation or containing preformed fibrils was also compared.^158^ Following a screening process, the catalytic amyloid Ac-IHIHIYI-NH_2_ was identified as being able, in the presence of Cu^2+^, to catalyze the hydrolysis of the toxic pesticide paraoxon.^159^ The catalytic efficiency *k*_cat_/*K*_M_ = 1.7 ± 0.6 M^–1^ min^–1^ is low compared to the performance of this peptide in pNPA hydrolysis *k*_cat_/*K*_M_ = 806 ± 100 M^–1^ min^–1^ in the presence of Cu^2+^ (*k*_cat_/*K*_M_ = 5888 ± 195 M^–1^ min^–1^ with Zn^2+^) (see also Table 1). The amyloid fibrils can be deposited onto a microfilter, and this can be used in a flow-through catalysis cell. This peptide can also catalyze cascade reactions such as the hydrolysis of 2′,7′-dichlorofluorescin diacetate (DCFH-DA), which produces 2′,7′-dichlorofluorescein (DCFH), which, in turn, is oxidized to produce highly fluorescent 2′,7′-dichlorofluorescein (DCF).^159^ In another example, Ac-IHIHIQI-NH_2_ was shown to catalyze the oxidation of 2,6-dimethoxyphenol (DMP) mediated by Cu^2+^, whereas nonfibril forming control peptide NH_2_-IHIHIQI-COOH (same peptide but uncapped) shows no catalytic enhancement compared to free copper ions.^160^

Dipeptides N-terminally functionalized with phosphanes that form antiparallel β-sheet structures serve as bidentate ligands that bind transition metal ions.^161^ In a proof of concept, these peptide conjugates were shown to enable rhodium(I)-catalyzed asymmetric hydroformylation reactions of styrene at high yield and with excellent regioselectivity but modest enantioselectivity.

Conjugating two amyloid peptide sequences, NADFDG from RNA polymerase (with low aggregation propensity) and a designed amyloidogenic sequence QMAVHV (with high aggregation propensity), leads to a peptide able (with Mg^2+^ or Mn^2+^) to catalyze ATP hydrolysis.^162^ The RNA polymerase sequence design was inspired by natural nucleotidyltransferases (NTases), which act on nucleotides via coordination of Mg^2+^ or Mn^2+^ ions to aspartate or glutamate residues. Metal-dependent fibril formation was a requirement for catalytic activity.

A pH-responsive “switch” peptide has been developed which exhibits esterase activity in its folded form at high pH, under which conditions the peptide forms β-sheet fibrils.^110^ The peptide is based on the “MAX” series of folded peptides^163^ which have a ^D^PPT turn structure which forms an ordered intramolecular β-turn under appropriate conditions. The peptide studied by Ulijn and co-workers folds at high pH and was functionalized with a histidine residue at the N-terminus (Table 1). This leads to histidine-functionalized fibrils under alkaline conditions. The catalysis was shown to be reversible when pH was switched from conditions of folded (for example pH 9) to unfolded (pH 6) conformation. A hydrogel forms under alkaline conditions at higher peptide concentrations and also shows catalytic activity.^110^

**Figure 8:**
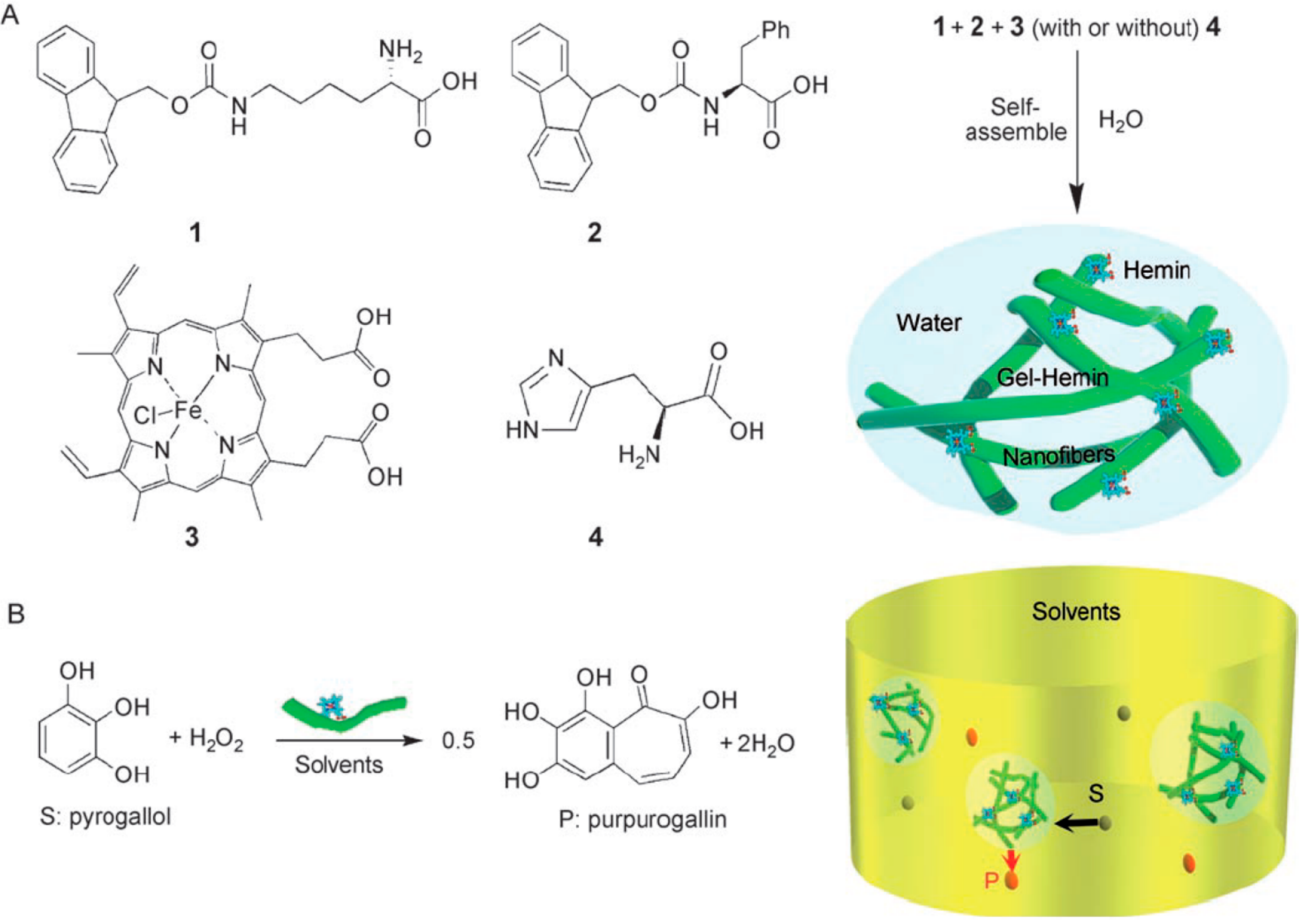
(a) Molecular structures of Fmoc-amino acids **1** and **2** coassembled with hemin chloride **3** (with or without histidine **4**) form hemin-loaded fibrils in a hydrogel. (b) The hydrogel can be used to catalyze the peroxidation of pyrogallol to purporogallin as shown.^164^ Reprinted with permission from ref (164). Copyright 2007 Wiley-VCH GmbH.

The simple Fmoc-phenylalanine [Fmoc: fluorenylmethyloxycarbonyl] molecule forms supramolecular fibrils when mixed with a Fmoc-ε-amino linked lysine derivative and hemin chloride in a hydrogel with catalytic peroxidase mimic properties, using the peroxidation of pyrogallol to purpurogallin with easy colorimetric detection (Figure 8).^164^ The catalytic activity is greater than that of free hemin, and in toluene the activity reaches about 60% of the nascent activity of horseradish peroxidase (HRP).^164^ Even the individual amino acid phenylalanine is able to aggregate in the presence of Zn^2+^ ions into “amyloid-like” needle-shaped crystals which exhibit catalytic esterase activity, as shown using pNPA as substrate (and monitoring by UV–vis absorption spectroscopy the appearance of yellow product, *p*-nitrophenolate, reaction **A**Scheme 1) and related derivatives.^104^ The activity was found to be substrate-specific and stereoselective. A Michaelis–Menten analysis of the hydrolysis kinetics provided *k*_cat_/*K*_M_, = 76.54 M^–1^ s^–1^,^104^ which is high compared to other peptide biomolecular hydrolases, as illustrated in Table 1, and although lower than carbonic anhydrase itself on a molar basis, it is better on a molality basis.

As well as nanofibrils, peptide nanotubes have been shown to exhibit catalytic activity. In one early example, nanotube-forming Fmoc-FFH-NH_2_ was shown to have catalytic activity as an esterase, although this could be enhanced by coassembling the peptide conjugate with small amounts of Fmoc-FFR-NH_2_.^111^ The authors noted that too large Fmoc-FFR-NH_2_ contents can lead to disruption of nanotube structure, which affects the ordered presentation of the histidine residues at the nanotube surfaces.^111^Table 1 lists the best catalytic efficiency observed for this system. Hydrogels are formed by Fmoc-FFH at sufficiently high concentration. This system was developed to produce cross-linked capsules by electrostatic complexation with PEI (polyethylenimine) followed by covalent cross-linking with glutaraldehyde.^165^ The capsules with imidazole group catalytic sites exhibit high catalytic activity for the hydrolysis of pNPA. The system shows a high degree of reusability (93% productivity retained after 15 cycles). The authors note that the hydrophobic microenvironment within the capsules enables this system to exhibit higher catalytic activity for the hydrolysis of pNPA compared to the Fmoc-FFH hydrogels.^165^ Later, a mimic of the catalytic triad H, S, D site in many natural hydrolases was designed based on coassembly of Fmoc-FFH, Fmoc-FFS, and Fmoc-FFD into fibrils.^166^ The system was studied as a catalyst for the model pNPA hydrolysis, and the hybrid coassembled system outperforms the Fmoc-FFH system with approximately doubled efficiency. As well as Fmoc-tripeptides, even nonconjugated tripeptides that are suitably functionalized can act as small peptide-based biocatalysts. For example, the tripeptide ^L^His–^D^Phe–^D^Phe forms β-sheet fibrils and forms a thermoreversible hydrogel in PBS buffer.^167^ This peptide shows significant catalytic activity, in terms of pNPA hydrolysis. A designed peptide bearing a histidine residue and an alternating β-sheet promoting sequence HSGQQKFQFQFEQQ-NH_2_ was shown to form fibrils and demonstrated esterase activity which was enhanced by coassembling this peptide with its arginine-bearing homologue with H1R substitution.^112^ It was proposed that the arginine guanidyl group stabilizes the transition state of the substrate at the binding site on the fibrils.

**Figure 9:**
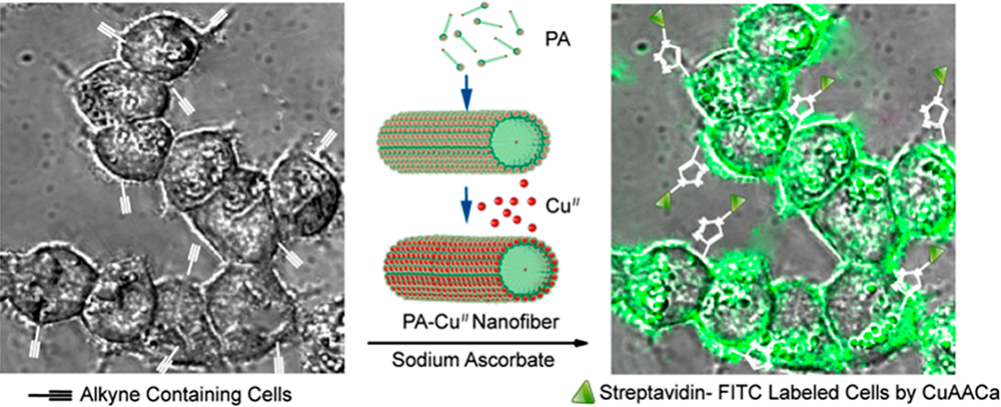
Fluorescent labeling of cells using copper-functionalized fibrils of a peptide amphiphile (PA) to catalyze a click reaction between alkyne-functionalized cell membranes (using an alkyne-functionalized mannose amine) and azide-biotin followed by streptavidin-FITC fluorophore noncovalent binding to the biotin groups.^168^ Reprinted with permission from ref (168). Copyright 2015 American Chemical Society.

Peptide amphiphiles (lipopeptides) that form nanofibrils have also been shown to catalyze ester hydrolysis, as exemplified in a study of HK_H_-LLLAAA(K)-palmitoyl [K_H_ denotes histidyl lysine, Scheme 3] and analogues lacking the palmitoyl chain or with LPPP replacing LLLAAA, the conformationally constrained proline sequence disfavoring β-sheet formation (Scheme 3).^108^ Indeed, the non-lipidated peptide and proline-substituted lipopeptides self-assembled into spherical micelles, for which a lower hydrolysis rate was observed than for nanofibril-forming HK_H_-LLLAAA(K)-palmitoyl (the value for the Michaelis–Menten catalytic efficiency for the hydrolysis of 2,4-dinitrophenyl acetate, DNPA, is included in Table 1).^108^ This points to the possibility to enhance catalytic activity using high aspect ratio fibril structures which also possess a high degree of molecular ordering including positioning of the HK_H_ catalytic residues which decorate the nanofibril surface. The catalytic H, S, D triad concept by coassembly was also used independently by Guler and co-workers, who explored the use of lipopeptides with these terminal residues, specifically mixtures of C_12_-VVAGX-NH_2_ (X = H, S, D) molecules.^109^ Co-assembly led to tapelike β-sheet nanostructures, and the three-component mixture showed better catalytic activity in pNPA hydrolysis (catalytic efficiency value reported in Table 1) and acetylcholine esterase experiments in comparison to two-component or single component systems. This group also showed that C_12_-VVAGHH-NH_2_ forms fibrils that can catalyze Cu^2+^ click reactions to fluorescently label live cells with streptavidin-FITC [FITC: fluorescein isothiocyanate] via reaction with cells bearing biotin-azide reacted with alkyne sialic acid chains in the membrane, as shown schematically in Figure 9.^168^ The morphology of lipopeptides C_16_-XYL_3_K_3_ (where XY = AA, AH, HH, MH) can be switched from micelles at pH 7 to fibrils at pH 10.5.^169^ These molecules are able to bind heme, and the heme active site is available in both micelles and fibers to bind carbon monoxide; however, unexpectedly peroxidase activity was observed in heme-containing micelles yet was significantly reduced in heme-containing fibers, which was ascribed to a reduced ability to generate reactive oxygen species.

**Scheme 3:**
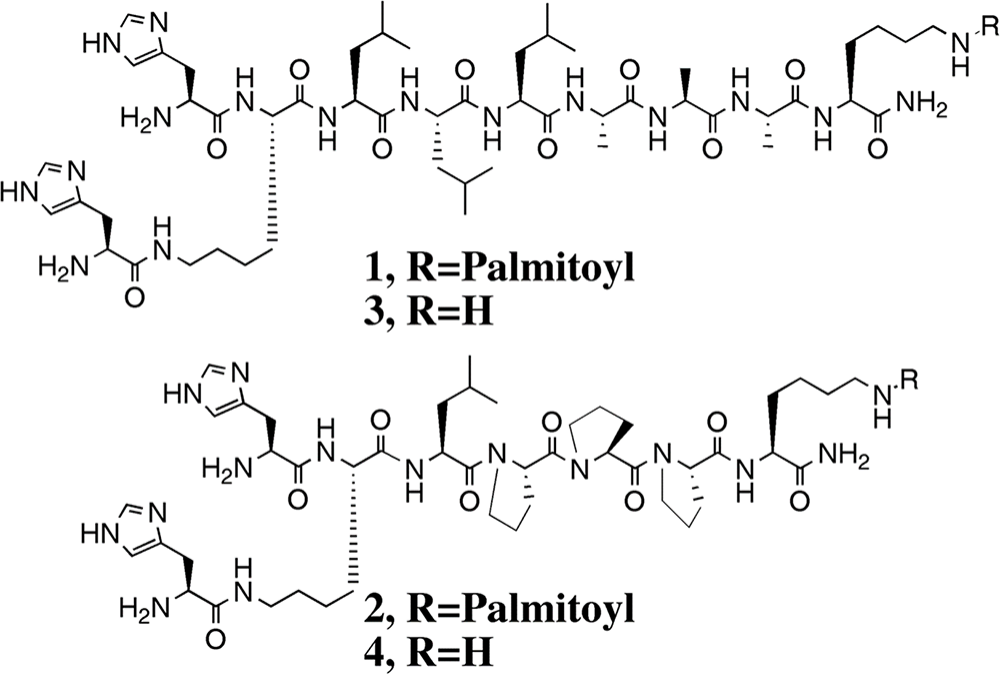
Lipopeptides and Controls Studied by Guler and Stupp^108^^,^ Molecule **1** forms β-sheet nanofibrils in contrast to non-lipidated control **3** whereas **2** and **4** are analogues with proline residues to disrupt β-sheet formation. Reprinted with permission from ref (108). Copyright 2007 American Chemical Society.

A range of histidine-functionalized lipopeptides including C-terminal peptide conjugates C_n_-GGH and C_10_-GSH and N-terminal HGG-C_n_ (n = 8, 10, 12) have been prepared (along with controls with glycine residues replacing histidines) and their self-assembly and catalytic properties examined.^170^ The lipopeptides self-assemble above a critical aggregation concentration into fibril structures (or globular aggregates, depending on the sequence). The histidine-functionalized molecules can catalyze pNPA hydrolysis (the efficiency is not reported, so the molecules are not listed in Table 1).^170^

Peptides containing alternating sequences including phenylalanine or isoleucine as hydrophobic residues, such as Ac-FEFEAEA-CONH_2_, were designed to favor β-sheet fibril formation.^171^ This peptide shows the highest activity (at pH 3 and 25 °C) among the series of related peptides studied, in terms of catalytic activity in the hydrolysis of cellobiose, a glucose dimer that is the simplest model glycoside for cellulose. Specificity was demonstrated since there was no activity against other disaccharides such as sucrose, maltose, or lactose. This peptide lacks a histidine residue, and the catalysis proceeds via a mechanism that does not involve imidazole units; but a tentative model based on the organization of the carboxylic acid units was proposed since a polymer (poly(acrylic acid)) with a disordered presentation of carboxylic acids showed no catalytic activity.^171^

**Figure 10:**
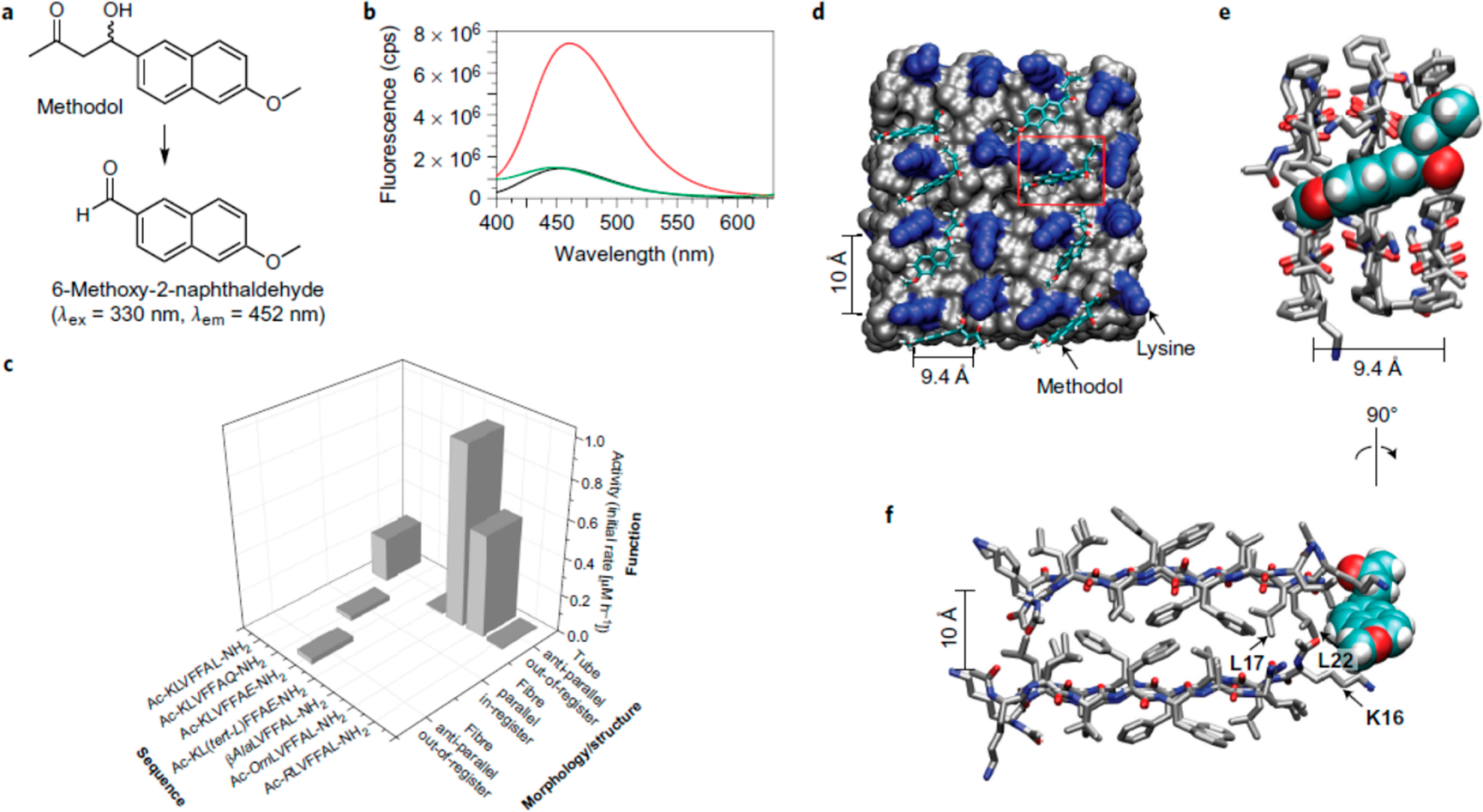
Retro-aldol catalysis using KLVFF-based peptide nanotubes. (a) Retro-aldol reaction of methodol to give 6-methoxy-2-naphthaldehyde, with indicated fluorescence peaks. (b) Fluorescence emission spectra (λ_ex_ = 330 nm) of 50 μM (±)-methodol (black line), 50 μM (±)-methodol with 1 mM Ac-KLVFFAL-NH_2_ nanotubes (red line), and 50 μM methodol with 1 mM Ac-RLVFFAL-NH_2_ nanotubes (green line). (c) Initial rate of production of 6-methoxy-2-naphthaldehyde by the indicated peptide assembly where the peptide concentration was 500 μM and the starting (±)-methodol concentration was 80 μM. (d), Molecular dynamics simulation of (*S*)-methodol docked onto the surface of Ac-KLVFFAL-NH_2_ antiparallel out-of-register amyloid assembly. In the space filling models the hydrophobic LVFFAL residues are colored gray, the lysines are blue, and methodol is drawn in a stick representation with carbons colored green, oxygen red, and hydrogen white. (e and f) Detail of arrangement of methodol (space filling) on tube surface with peptides drawn as sticks. Reprinted by permission from Springer-Nature: Nature Chemistry, ref (175), Copyright 2017.

The amyloid β (Aβ) peptide (in both Aβ40 and Aβ42 form) that forms fibrils and is implicated in Alzheimer’s disease is able to bind heme, and the bound complex has peroxidase activity.^172,173^ The authors of these studies proposed that Aβ binding to regulatory heme is actually the mechanism by which Aβ causes heme deficiency *in vivo* and also heme binding hinders Aβ aggregation. The complex catalyzes the oxidation of serotonin and 3,4-dihydroxyphenylalanine by hydrogen peroxide.^173^ Lynn’s group exploited the KLVFF motif (the sequence Aβ(16–20) of the Aβ peptide^174^) in peptide derivatives; in particular, they showed that Ac-KLVFFAL-NH_2_ can catalyze the retro-aldol reaction of methodol to 6-methoxy-2-naphthaldehyde.^175^ This peptide forms nanotubes in the studied water/acetonitrile mixture, with the nanotube walls comprising antiparallel β-strands. The authors also examined the activity of homologues with different cationic side chain lengths, replacing the lysine residue with ornithine, arginine, and other derivatives.^175^Figure 10 shows the details of the methodol aldol reaction along with the fluorescence spectra used to detect the product and representative initial rate data for several derivatives and a model of the nanotube surface showing the simulated arrangement of methodol molecules within the β-strand array at the nanotube surface.

**Figure 11:**
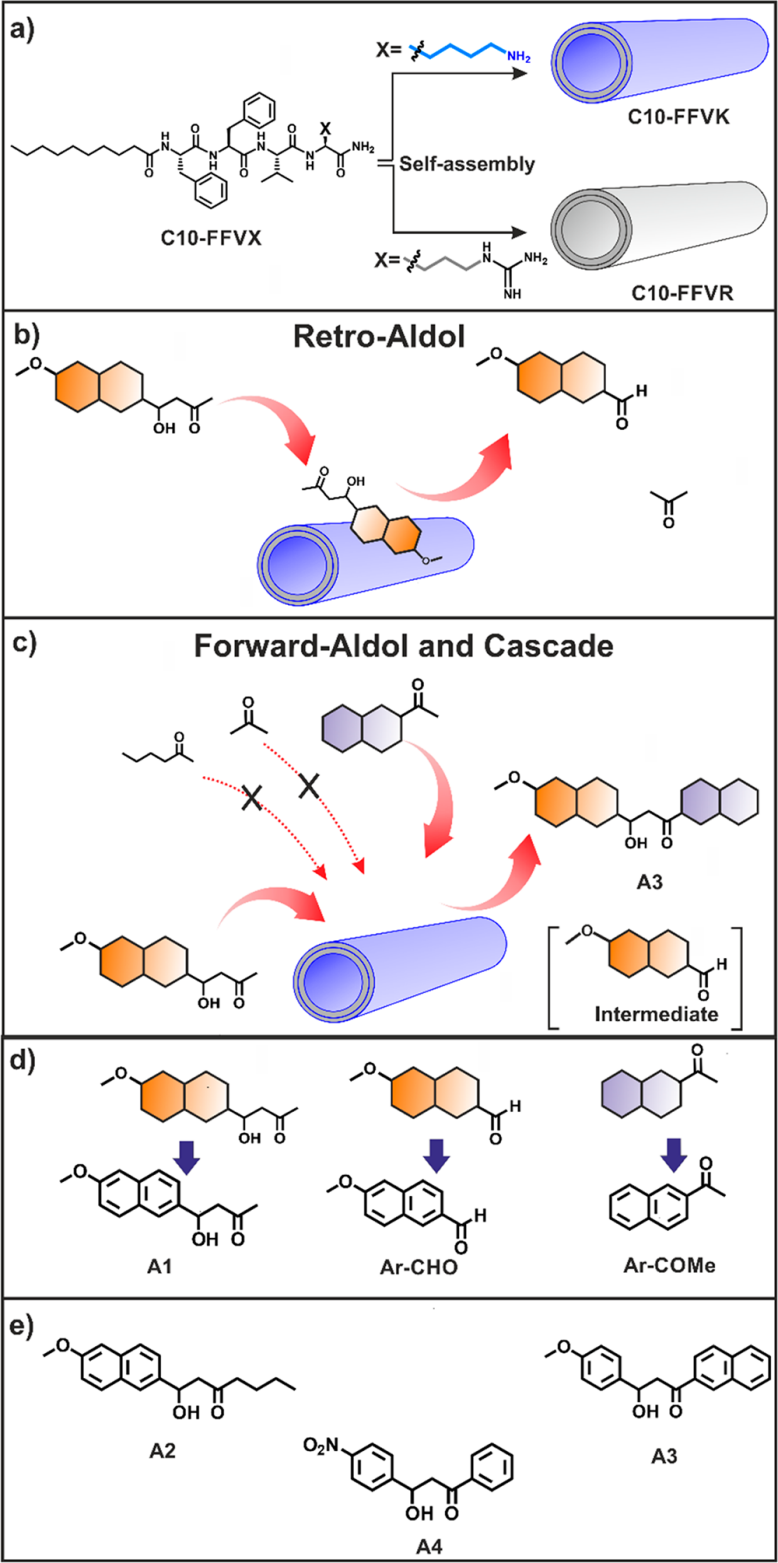
Lipopeptide nanotubes catalyze aldol reactions. (a) Lipopeptides based on the VFF sequence from the Aβ peptide with cationic residues X shown. (b and c) Aldol reactions in catalysis studies. (d and e) Substrates and products. Reprinted with permission from ref (176). Copyright 2020 Wiley-VCH GmbH.

Recently, the Das group has developed KLVFF-based nanotube peptide catalysts in a number of impressive directions and have demonstrated catalysis of aldol and retro-aldol reactions by KLVFF-based peptides and lipopeptide^176^ nanotubes and hydrolase activity of XLVFF-based nanotubes functionalized with cationic residues X and imine-promoting functionalities.^177^ Lipopeptides C_10_-FFVX-NH_2_ where X is lysine or arginine (Figure 11a) show activity in catalyzing the retro-aldol, forward aldol, and cascade (combined bond breaking and formation) reactions shown in Figure 11b and c (Figure 11d and e show the detailed molecular structures of substrates and adducts).^176^ The retro-aldol reaction converts methodol (A1) to 6-methoxy-2-naphthaldehyde (Ar-CHO) as in the work of Lynn and co-workers (Figure 10a). The retro-aldol activity was much higher for C_10_-FFVK than C_10_-FFVR, and A1 was cleaved with a much higher rate constant than the homologue A2 (Figure 11e); this may be due to the formation of imine intermediates. The authors also showed the enhancement of activity of nanotubes compared to fibrils (which are kinetic intermediates formed in the assembly of C_10_-FFVK). The fibrils also showed lower selectivity for cleavage of A1 compared to A2. In a last step, the authors conjectured that activity would be enhanced by incorporation of tyrosine as in existing aldolases which contain this residue in the active site, acting as a hydrogen bond donor. They used mixtures of nanotubes with Fmoc-Y which binds to the nanotube surface. A significant enhancement of activity was indeed observed in the presence of this tyrosine derivative, consistent with the authors’ hypothesis.^176^

**Figure 12:**
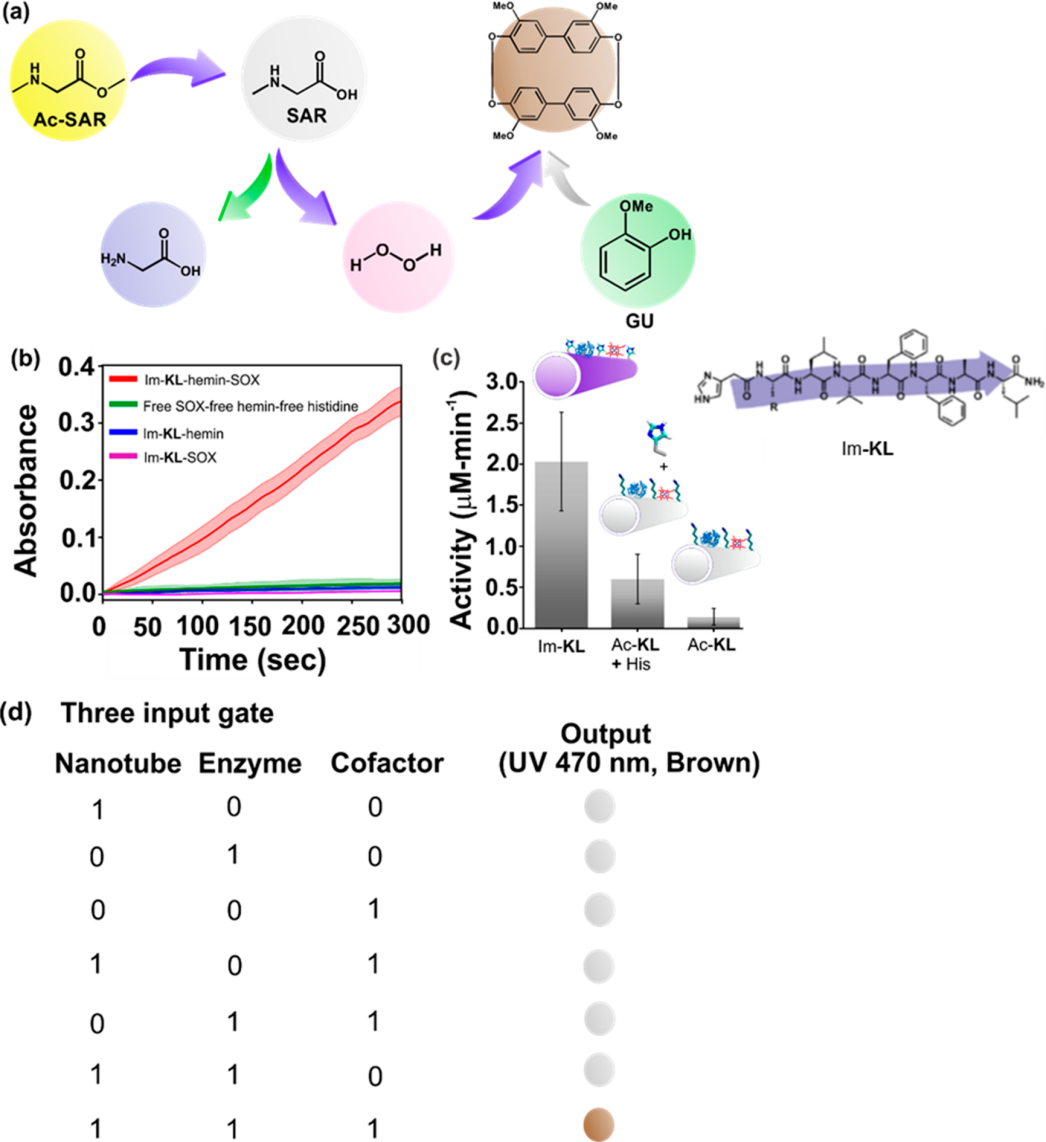
Three-step cascade reaction leading to the development of a three-input AND logic gate. (a) Schematic of reactions. (b) Kinetics of product formation for the indicated species. (c) Bar diagram of activity of the Im-KL nanotubes (molecular structure shown in the inset along with β-strand indication) along with controls lacking the imidazole N-terminus, i.e. Ac-KL and Ac-KL with histidine. (d) Three-input Boolean logic gate, with output only in the case of all three inputs. Reprinted with permission from ref (178). Copyright 2021 Wiley-VCH GmbH.

An N-terminal imidazole-functionalized KLVFFAL peptide (called Im-KL, Figure 12) that forms β-strand-based nanotubes has been used to create a model hydrolase.^177^ The nanotube surfaces facilitate Schiff imine formation via the exposed lysines to efficiently hydrolyze activated and inactivated esters. This was confirmed using control samples with substitutions of the K residue for R, O (ornithine), or E. The activity was higher for substrates containing a keto group such as keto ester 4-nitrophenyl 4-oxopentanoate. The lower activity of the variant with a lysine-ornithine substitution was ascribed to lower accessibility of the amine groups, which was modeled in terms of the packing at the nanotube surface. A control system comprising Ac-KL with added imidazolic acid showed greatly reduced hydrolase activity, pointing to the necessity of an array of both imidazole and lysine amine groups to enable the imine formation and hence catalytic activity.^177^

The same Im-KL peptide has been investigated in two-step, three-step, and convergent cascade catalysis reactions.^178^ The two-step cascade reaction uses sarcosine oxidase (SOX) and a prosthetic hemin group with peroxidase activity, both of which bind to the peptide nanotube walls. The substrate sarcosine is demethylated to give glycine, formaldehyde, and hydrogen peroxide which fuels the second reaction, with guaiacol (GU in Figure 12a) as substrate. The three-step and convergent cascade reactions include a first step starting from methylated sarcosine which is hydrolyzed by the imidazole functionalized peptide nanotubes (Figure 12). The demonstrated cascade reaction activity led to the creation of a three-input AND logic gate as shown in Figure 12d where the inputs are the peptide nanotubes, the enzyme (SOX), and the cofactor (hemin), and the output is a brown product (Figure 12d) detected by absorbance at 470 nm. A multigate logic network with several AND gates was also demonstrated.^178^

Researchers have investigated histidine-containing β-sheet fibril-forming peptides for other catalytic reactions including hydrolysis of amide bonds (amidolysis).^179^ Among a series of related peptides containing the H, S, D catalytic triad, Ac-FFSGHFDFF-NH_2_ and Ac-FGFHFSFDF-NH_2_ were shown to form β-sheet-based fibrils and the latter shows pH- (and temperature-) dependent hydrolytic catalytic activity using l-alanine p-nitroanilide as substrate.^179^

The β-sheet forming histidine-based analogues of the proline-functionalized peptide bola-amphiphiles discussed in section 3 have been studied, and these show activity in the catalysis of ester hydrolysis.^113^ For example, HV-C_8_-VH (**5** in Scheme 2) forms gels based on fibrils or nanotapes, depending on pH, and catalyzes pNPA hydrolysis, the catalytic efficiency at pH 7 being shown in Table 1 (with comparable values in the range pH 6–8). The control peptide H-C_8_-H does not aggregate and shows much lower catalytic efficiency.^113^ An analogue PyrV-C_3_-VPyr [Pyr: pyridyl] (**6** in Scheme 2 and the 3-pyridyl analogue) can form fibrillar organogel structures and complexes with Pd^2+^, and the gel fibrils are able to catalyze aerobic oxidation of benzyl alcohol.^180^ A related conjugate H-C_7_-H with Zn^2+^ catalyzes CO_2_ hydration and serves as a carbonic anhydrase mimic.^181^ Sequestration of CO_2_ leads to the precipitation of CaCO_3_ upon the addition of Ca^2+^ ions. This histidine bola-amphiphile self-assembles into globular structures. The hydrolysis of pNPA was also analyzed, although the reported maximal catalytic efficiency *k*_cat_/*K*_M_ = 1.32 M^–1^ s^–1^ (at pH 7, 25 °C) is not high compared to many other systems (Table 1).^181^ This reaction was monitored in a development in which a Y-C_7_-Y analogue was used to detect (by photoluminescence quenching of this tyrosine bola-amphiphile) the product of pNPA hydrolysis (**D** in Scheme 1) by H-C_7_-H.^182^ The possible coassembly of these peptide conjugates was not investigated.

Nanotubes are formed by the peptide bola-amphiphile *N*,*N*′-hexadecanedioyl-di-l-glutamic acid (L-HDGA) (and related compounds), and complex formation with Cu^2+^ leads to a transition from single to multilayer nanotubes.^183^ The system is able to form a hydrogel. The self-assembly of related l-glutamic acid derivatives into nanotubes was not stable to Cu^2+^ addition. The L-HDGA system was able to catalyze Diels–Alder cycloaddition reactions with high yield and diastereoselectivity but rather low enantiomeric excess values.

## 5 Peptide Catalysts with Metal Nanoparticles

Screening of libraries of peptides for biocatalytic activity using phage display, combinatorial approaches using arrays of bead-tethered peptides etc., has been the subject of a number of studies reviewed elsewhere.^184,185^ These are not discussed in detail here as they do not focus on the use of self-assembled or other ordered peptide structures. In an approach where the peptide was catalytically active when attached to a filamentous phage, screening (biopanning) of large numbers of peptides for catalytic activity used phage display to produce a random library of 10^9^ dodecameric peptide sequences.^186^ The peptide repeats were displayed at the tip of filamentous M13 phages, and catalytic activity was assayed for hydrolysis reactions of peptides and pNPA and an amide coupling reaction, all under physiological conditions. No apparent sequence homology was in the peptide sequences obtained after biopanning (which contained H, S, D, E, C residues associated with nucleophilic reactions), indicating that function can be found in random peptide sequences.^186^ The catalytic activity for pNPA hydrolysis could be significantly enhanced by conjugation of one of the identified catalytic sequences to a β-sheet fibril forming peptide (FFKLVFF) based on the core Aβ peptide sequence KLVFF.^187^ This confirms the important role of organized presentation of the catalytic S, H, E triad along with hydrophobic stabilization of the bound substrate.^187^ The same peptide (SMESLSKTHHYR) was also shown to be able to catalyze the growth of ZnO nanocrystals at room temperature from zinc acetate precursor solution, i.e. via reverse ester hydrolysis.^188^ Studies on mutants showed the importance of the S–K dyad which is also found in proteases. Another approach to the conjugation of peptides to elongated scaffolds involved the attachment of mixtures of peptides SHELKLKLKL and WLKLKLKL to carbon nanotubes; the former contains the catalytic S, H, E triad, and the latter contains a tryptophan binding domain.^117^ These alternating peptides would be expected to form β-sheets, although α-helical CD spectra were assigned by the authors. The catalysis of the hydrolysis of pNPA was reported with the C-terminal SHE-catalytic triad linked to the carbon nanotube (Table 1 lists the catalytic efficiency), with a lower efficiency when the N-terminal L residue of SHELKLKLKL was attached. This points to the enhancement of catalytic activity by the hydrophobic carbon nanotube surface environment.^117^

**Figure 13:**
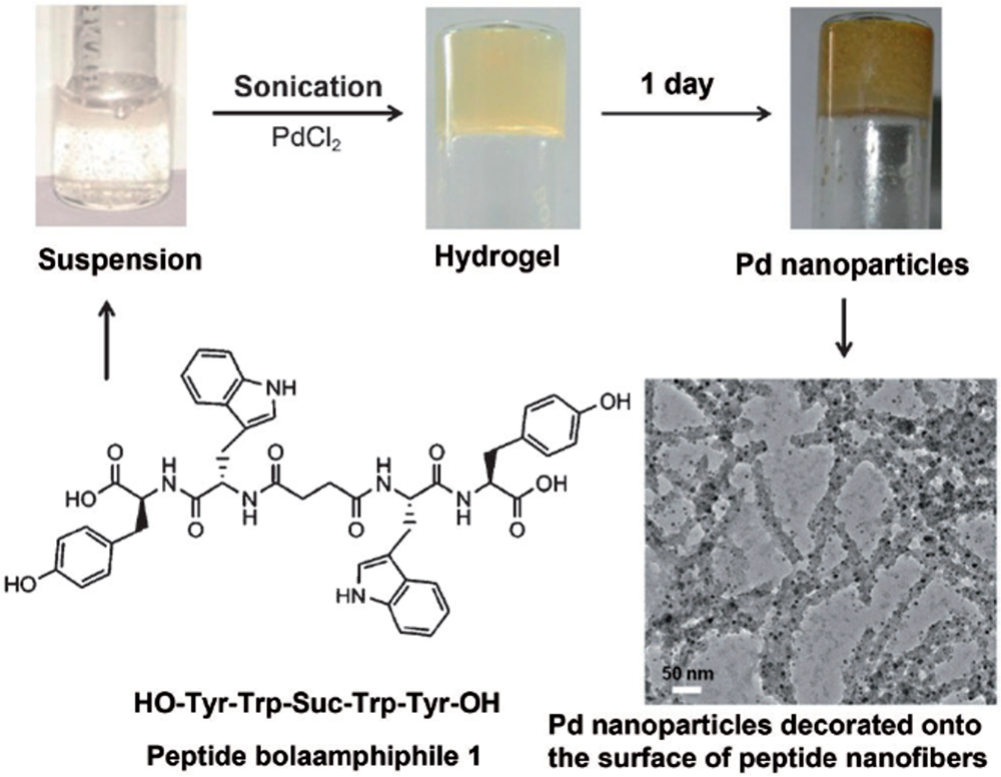
Molecular structure of peptide bola-amphiphile studied by Maity et al. along with images of solution and hydrogels in vials including a hydrogel containing Pd nanoparticles and a TEM image of Pd-decorated peptide fibrils.^194^ Reprinted with permission from ref (194). Copyright 2014 Wiley-VCH GmbH.

Peptides can be used as templates to control the shape, size, faceting, orientation, and composition of noble metal catalysts such as Au, Pd, Pt, CdS, etc. This topic is reviewed in more detail elsewhere.^189,190^ In addition to their structuring role, templating peptides can cap the noble metal nanoparticles and can boost catalytic performance. Another route to the enhancement of catalytic activity that has been explored is to decorate peptide nanostructures such as amyloid fibrils with catalytic metal particles. In one example, an N-terminal aniline functionalized amyloid peptide GGAAKLVFF forms positively charged fibrils which were used to adsorb negatively charged citrate-functionalized Pt nanoparticles.^191^ The electrocatalytic activity of the Pt–peptide fibrils in terms of oxygen reduction was then investigated, and this system showed higher electrocatalytic activity than that of several other Pt-based nanomaterials. Other examples include the extension of the work of Guler’s group discussed in section 4 with lipopeptide C_12_-VVAGHH-NH_2_. They prepared Pd nanoparticles by reduction of Pd^2+^ in the presence of the peptide fibrils (rather than using preformed Pd nanoparticles added to the peptide fibril solution).^192^ The resulting Pd-functionalized peptide fibrils showed excellent catalytic activity for Suzuki–Miyaura coupling of aryl iodides. Using a similar concept, nanofibrils formed by the surfactant-like peptide I_3_K were used as a template for Pt deposition.^193^ Platinum salt precursors were immobilized by electrostatic interaction on the positively charged fibrils, and subsequent reduction led to the production of one-dimensional Pt nanostructures. The electrocatalytic performance of the Pt-functionalized fibrils in hydrogen and methanol electro-oxidation was examined. Palladium nanoparticles supported on fibrils of the peptide bola-amphiphile YW-Suc-WY [Suc denotes a succinic-acid-based linker] were shown to have catalytic activity for the deprotection of N-terminal groups of several amino acids and peptides.^194^Figure 13 shows the peptide sequence along with a TEM image of Pd-decorated fibrils and images of vials containing solution and gels. Naturally derived peptide R5 (from a diatom cell wall) with sequence KKSGSYSGSKGSKRRIL forms spherical nanoparticles, nanoparticle clusters, or nanoribbons in the presence of precursor Au^4+^ or Pt^2+^ salts followed by reduction.^195^ These structures were used in the electrocatalysis of oxygen reduction. A cobalt-binding peptide was incorporated in a conjugate BP-PEPCo (C_12_H_9_CO-HYPTLPLGSSTY) [BP: biphenyl] which forms hollow spherical nanostructures with a shell of CoPt nanoparticles.^196^ Electrochemical measurements were performed of the catalysis of methanol oxidation in the presence of these nanoparticle superstructures.

It has been shown that catalytic triads from serine proteases (H, S, and D residues) that have been incorporated in a designed lipopeptide-like molecule lead to an effective hydrolase.^197^ The peptide surfactant coassembles with conventional cationic surfactants into spherical micelles. Other researchers have explored the use of peptides as agents to bind and template nanoparticles such as gold and silver nanoparticles with a diversity of catalytic properties.^198^

The use of conjugates of peptides, for example PEG-peptides, has been explored for metal nanoparticle production. For example, PEG-polytyrosine conjugates were used to produce gold nanoparticles by reduction of HAuCl_4_ precursor salt solution.^199^ Different self-assembled structures were observed for the PEG-polytyrosine molecules depending on the hydrophobic polytyrosine chain length (the PEG chain length was fixed at n = 43) including fibrils, vesicles, and spherical nanoparticles. The chloroaurate binding capacity, and hence gold nanoparticle formation, was also reported to depend on the conjugate composition. The peptide conjugate-templated gold nanoparticles showed activity as catalysts of 4-nitrophenol reduction, with the observed rate constant being compared to that of other gold nanoparticle-based catalysts.^199^ Complexes of peptides with other molecules have also been explored as biocatalysts. In one example, a polycation, H_32_ (32 repeats of histidine), undergoes electrostatic complexation with anionic DNA, specifically guanine-rich DNAzyme-I.^200^ Incorporation of hemin into the complexes leads to peroxidase-mimicking nanoparticles. Catalyzed oxidization of ABTS^2–^ [ABTS: 2,2′-azinobis(3-ethylbenzothiazoline-6-sulfonic acid)] in the presence of hydrogen peroxide was studied by monitoring the time-dependent absorbance changes of the product, ABTS^•+^.

## 6 Conclusions

This Review shows the considerable potential that self-assembled peptides have in the catalysis of many types of reactions in organic chemistry. Presentation of catalytic motifs at the surface of ordered structures including micelles, fibrils, nanotubes, or coiled coil bundles can facilitate catalysis due to the persistent high density functionality of the nanostructures. In addition, well-ordered (“rigid”) peptide structures such as those based on α-helices and nanotubes can be used to precisely position catalytic residues such as those in important catalytic triads, with this mimicking to a certain extent the positioning of residues due to folding in natural enzymes. This can of course more closely be matched by protein engineering methods or the design of mini-proteins, although this Review has focused on nanostructures formed by shorter peptides. The development of these for applications in catalysis offers advantages compared to larger proteins in ease of design (including high-throughput sequence screening, utilization of known rules of assembly of α-helical and β-sheet peptides, etc.), cost effectiveness, scale-up (via automated peptide synthesis or recombinant expression), and the possibility to engineer enhanced stability against pH, temperature, etc., avoiding problems with inactivation or unfolding of natural enzymes. Assembly rules for peptide coiled coils^12−19^ (and to a lesser extent amyloid β-sheet systems, based on aggregation propensity^20,201−204^) enable a bottom-up minimalist approach for catalyst design. It is interesting that certain peptide structures such as some amyloids also show autocatalytic properties, reviewed elsewhere.^57^ Peptide hydrogels and organogels based on fibrillar structures also show good catalytic performance, and reversible sol–gel transitions can be used as a recycling method.

Among natural residues, proline and histidine are particularly useful in promoting catalytic activity in peptide nanostructures. Although both contain five-membered amine rings, there are clear differences in that proline contains an aliphatic pentameric secondary amine ring, whereas histidine contains an aromatic imidazole group which when unprotonated is nucleophilic and acts as a base with two tautomeric forms. In its protonated form (below the p*K*_a_ of approximately 6.0) the imidazolium group is positively charged. An important property of histidine is its ability to bind metal ions and metal complexes such as heme, and this and zinc-dependent catalytic activity have been widely explored as discussed above in peptide peroxidase mimics and others.

The acid–base properties of the imidazole side chain of histidine underpin the catalytic activity of histidine-based peptide nanostructures, and they play an essential role in the activity of the H, S, D catalytic triad (and closely related H, S, E triad) present in many proteases. This motif has also been incorporated in peptide nanostructures via coassembly of different peptides and peptide conjugates including amyloid peptides, lipopeptides, and Fmoc-tripeptides forming fibrils. The related H, C, E triad has been incorporated into coiled coil assemblies and the H, S, E triad has been used in peptides tethered to carbon nanotube supports. Triads of cysteine residues and/or of histidine residues have been incorporated into coiled coil bundles to facilitate metal ion binding.

Decoration of peptide nanostructures, especially fibrils, with catalytically active metal nanoparticles has already been demonstrated as highlighted by the examples discussed in section 5. This also offers possibilities to develop catalysts for other reactions especially oxidation and reduction reactions including those associated with industrially important processes.

Future research is likely to lead to enhanced performance of peptide catalysts by optimizing the stability of the nanostructures (covalently or noncovalently) as well as more accurate positioning of residues, with input from state-of-the-art computational modeling of the binding sites, transition states, and catalysis mechanisms. More stable peptide catalysts could also expand the range of reactions that could be catalyzed under more extreme conditions (at the moment there are limitations on the stability of peptide structures to extreme pH, temperature, or the presence of certain organic solvents etc.). New high-throughput peptide screening methods and other advances in synthesis techniques, for example genetic expression technologies allowing incorporation of catalytically active non-natural residues^205^ (or synthetic methods to achieve this), new coupling chemistries for novel conjugate design, and so forth, also hold great promise for discoveries directed toward the enhancement of catalytic properties and the range of reactions that can be catalyzed. Improving the reusability and recyclability of peptide catalysts is also a challenge for future research. Related to the research on peptide gels discussed above, peptide functionalization of other porous media (porous monoliths and membranes for instance) is also a promising area for further research, as it will permit flow-through catalysis reactions relevant to industrial processing. Investigating the use of peptides to catalyze complex cascade reactions and to develop biologic gates (to coin a phrase) as exemplified for example in the recent work by Das’ group mentioned above^178^ is also likely to be a very fruitful avenue of research. The promotion of coupling reactions of biomolecules by peptides is also intriguing. This will see greater interest, since it enables the use of biocompatible catalysts containing the functional diversity of peptide residues, as shown by the example of the work of Guler’s group demonstrating the catalysis of *in vivo* coupling reactions using live cells^168^ mentioned above.

Clearly, with their diversity of programmable functions and unique properties in the development of biomimetic (enzyme-mimetic) structures, peptides offer outstanding potential in the creation of next-generation catalysts. Furthermore, peptides are biocompatible materials, and this gives the additional benefit that they can be used to produce environmentally friendly/green biocatalysts.
